# Changes of microorganism composition in fresh and stored bee pollen from Southern Germany

**DOI:** 10.1007/s11356-021-13932-4

**Published:** 2021-04-23

**Authors:** Carolin Friedle, Paul D’Alvise, Karsten Schweikert, Klaus Wallner, Martin Hasselmann

**Affiliations:** 1 Apicultural State Institute, University of Hohenheim, Stuttgart, Germany; 2 Institute of Animal Science, Department of Livestock Population Genomics, University of Hohenheim, Stuttgart, Germany; 3 Core Facility Hohenheim and Institute of Economics, University of Hohenheim, Stuttgart, Germany

**Keywords:** Microorganism composition, Bee pollen, Pollen quality, Pollen spoilage

## Abstract

Analysis of plant pollen can provide valuable insights into the existing spectrum of microorganisms in the environment. When harvesting bee-collected pollen as a dietary supplement for human consumption, timely preservation of the freshly collected pollen is fundamental for product quality. Environmental microorganisms contained in freshly collected pollen can lead to spoilage by degradation of pollen components. In this study, freshly collected bee pollen was sampled at different locations and stored under various storage conditions to examine the hypothesis that storage conditions may have an effect on the composition of microorganisms in pollen samples. The samples were analyzed using 16S and 18S amplicon sequencing and characterized by palynological analysis. Interestingly, the bacterial communities between pollen samples from different locations varied only slightly, whereas for fungal community compositions, this effect was substantially increased. Further, we noticed that fungal communities in pollen are particularly sensitive to storage conditions. The fungal genera proportion *Cladosporium* and *Mycosphaerella* decreased, while *Zygosaccharomyces* and *Aspergillus* increased during storage*. Aspergillus* and *Zygosaccharomyces* fractions increased during storage at 30 °C, which could negatively impact the pollen quality if it is used as a dietary supplement.

**Supplementary Information:**

The online version contains supplementary material available at 10.1007/s11356-021-13932-4.

## Introduction

The existing spectrum of microorganisms in the environment can be revealed in pollen samples from plants (Anderson et al. 2013; Manirajan et al. 2019). Pollen, the male gametophyte of gymnosperms and angiosperms, represents the main protein and lipid source for honey bees (*Apis mellifera*). It provides a full spectrum of not only nutrients, mainly amino acids and lipids, but also carbohydrates, minerals, vitamins, and enzymes (Feás et al. 2012; Pascoal et al. 2014; Avni et al. 2014; Kostić et al. 2015). Bee-collected pollen consists of pollen grains from flowering plants (including bushes and trees), as well as nectar and salivary secretion from the bees. It is collected by foraging honey bees and transported to the hive in the pollen baskets known as corbicula on their hind legs (Kevan and Baker 1983; Willmer 2011). After storage and microbe-mediated maturation in honeycomb cells, pollen is called “bee bread” and is consumed by nurse bees to produce protein-rich larval food supported by secretions of specialized glands (Lindauer 1952; Cridge et al. 2015). Bee pollen is perceived as a useful dietary supplement for humans, as it provides many necessary nutrients, especially a high amount of protein (± 20%) including essential amino acids like leucine, isoleucine, and valine, depending on the botanical origin (Paramás et al. 2005; Carpes et al. 2009; Martins et al. 2011; Feás et al. 2012; Nicolson and Human 2013; Taha et al. 2019).

The nutrient composition and microbiological quality of bee pollen depend strongly on its geographic and botanical origin, the weather at the time of collection, as well as on the post-harvest processing procedure by the bee keeper (Hani et al. 2012; Nogueira et al. 2012; Corby-Harris et al. 2014; De-Melo et al. 2015, 2016; Dinkov 2016; Disayathanoowat et al. 2020). When gathering of the pollen is not followed by drying or another processing step, growth of microorganisms can compromise pollen quality, leading to negative side effects like fermentation or mycotoxin production (González et al. 2005; Hani et al. 2012; Fatrcová-Šramková et al. 2016). The taxonomical identity of the identified microorganisms in fresh bee pollen suggests that the initial microbial community composition is rather arbitrary and may change in composition to more opportunistic microorganisms with unwanted characteristics that grow well under warm storage conditions (González et al. 2005; Hani et al. 2012). Following the hypothesis that storage conditions may impact the composition of microorganisms, resulting in the spoilage of pollen, we measured the differences in the microorganism composition of freshly harvested bee pollen and compared it with the same pollen after storage under different temperature treatments. This experimental approach aimed to identify the most relevant microorganisms that cause spoilage and identify the most favorable storage condition for preserving pollen quality during and after the harvest.

Previous studies have used cultivation-dependent methods, e.g., bacterial and fungal colony counts on agar plates (Bonvehí and Jordà 1997; González et al. 2005; Estevinho et al. 2012; Feás et al. 2012; Bárbara et al. 2015; De-Melo et al. 2015; Dinkov 2018), and cultivation-independent methods, such as 16S-rRNA amplicon sequencing to characterize the microbial composition in fresh, dried of frozen bee pollen to evaluate the risk of microbial hazards (Anderson et al. 2014; Corby-Harris et al. 2014; Mauriello et al. 2017; Moreno Andrade et al. 2018).

Recently, Disayathanoowat et al. (2020) analyzed the abundances of bacterial and fungal communities in fresh bee pollen and in “bee bread” that was stored for 72 h within the hive using amplicon sequencing and plate counting. They showed that abundance of bacteria was declining, while the fungal population remained at stable numbers during in-hive storage. However, to our knowledge, there is no information available about changes in bacterial and fungal communities in pollen being stored outside of the hive under defined conditions. Furthermore, taxonomic information about changes in the microbial communities of stored pollen are missing. Consequently, the present study was designed to assess the qualitative changes by next-generation sequencing of 16S and 18S PCR-amplicons in the microbial communities of pollen. Samples were stored under different conditions, to simulate how the composition of the microorganisms can be affected by wrong processing or non-harvesting of pollen.

## Materials and methods

### Sampling

Bee pollen was collected on 1 day in the month of June in 2 consecutive years at three different locations: Baden-Wuerttemberg, Southern Germany (Hohenheim in 2018 (H), Forbach in 2019 (F), and Nuertingen in 2019 (N)) (Figure S1). To minimize the presence of any potential microorganism on the pollen traps before, the traps were cleaned intensively with 70% ethanol and installed in front of one hive per site to collect pollen loads from returning honey bees (*Apis mellifera*) (Detroy and Harp 1976). The freshly collected pollen was divided at the same day of collection and allocated to four different groups “fresh,” “cold,” “room temperature,” and “warm.” Condition “cold” simulated a storage in the refrigerator and “room temperature” a storage without refrigeration after harvesting. The condition “warm” simulated unharvested pollen in the trap or pollen was left on environmental conditions of a hot summer day. The samples representing each group were further divided into triplicate samples of 3 g and filled into 2 mL tubes (VWR International, Bruchsal, Germany). Group “fresh” was stored immediately at − 80 °C until extraction, while the other groups were incubated at different temperatures for 7 days (168 h) and thereafter stored at − 80 °C until extraction. Group “cold” was incubated at cold temperatures (4 °C), group “room temperature” was incubated at 25 °C, and group “warm” was incubated at 30 °C and 75% humidity (humidity was adjusted with a saturated sodium chloride solution). All samples were incubated with open lids in humidity chambers (Figure S2) and samples of the groups “warm” and “room temperature” were further incubated in a warming cabinet (Binder, Tuttlingen, Germany) and samples of group “cold” were placed in a refrigerator (Siemens, Munich, Germany).

Pollen samples in Hohenheim were collected directly by the authors; samples from Forbach and Nuertingen were collected with the help of voluntary beekeepers. All samples were received from privately owned bee colonies, so no exact grid references are given, and no permits were needed for this study.

### Palynological analysis

A palynological analysis of all pooled samples of each location was performed. All pollen samples were mechanically homogenized by using a mortar, followed by weighing 100 mg homogenized pollen in a 50-mL tube (Buddeberg, Mannheim, Germany) containing 10 mL demineralized water and a drop of dish soap (Friedle et al. 2021). For each sample, 500 pollen grains were determined. Pollen morphology was identified using a light microscope (10 × 40; VWR International, Bruchsal, Germany).

### DNA extraction

To analyze the microbial community, DNA was extracted from all 36 bee pollen samples using a TRIzol protocol (D’Alvise et al. 2018; Seeburger et al. 2020). An aliquot of 50 mg pollen was weighted in to a 2 mL lysis tube with 50 μL 0.1 mm glass/zirconia beads and 500 μL TRIzol (Invitrogen, ThermoFisher Scientific, Schwerte, Germany). The samples were homogenized in a bead beater (FastPrep-24, MP Biomedicals, Eschwege, Germany) at 5.5 ms^−1^ for 50 s and incubated for 5 min at room temperature (RT). After adding 100 μL chloroform, shaking for 15 s, and further incubation for 5 min at RT, two phases were separated by 15 min centrifugation at 12,000×*g* and 4 °C. The aqueous phase was transferred to another tube for RNA extraction, which can be used for further experiments. To the other phase, 250 μL back extraction buffer (4 M guanidine thio-cyanate, 50 mM sodium citrate, 1 M TRIS) were added and extracted by shaking for 15 s. After 10 min incubation at RT and centrifugation (as before), the aqueous supernatant was transferred to a new tube and precipitated by mixing with 200 μL isopropanol, followed by centrifugation (as before). The supernatant was discarded, and 500 μL 75% ethanol were added to wash the sediment. After short centrifugation (5 min; 2000×*g*; 4 °C), the supernatant was removed and the sediment dried for 10 min at RT. An aliquot of 50 μL 8 mM NaOH were added to redissolve the sediment and the solution was centrifuged for 10 min at 12,000×*g* at RT to remove membrane lipids. The supernatant was transferred to a new tube and mixed with 4.25 μL 0.1 M HEPES and 0.5 μL RNAse A (10 mg ml^−1^ Amresco). The DNA extracts were then incubated for 1 h at 37 °C and stored at −20 °C until analysis. The DNA concentrations were determined using Qubit fluorometer (Thermo Fisher Scientific, Schwerte, Germany) and showed concentrations < 1 ng/μL. Some of the DNA extracts requiring additional purification were thereafter precipitated with absolute ethanol; pH was adjusted to 5.5 with 5 μL 3 M sodium acetate solution, then the sample was mixed with 125 μL cold ethanol (absolute). After incubation (15 min at 4 °C) and centrifugation (20 min; 17,000×*g*; 4 °C), the supernatant was removed, the sediment was dried for 5 min at RT and dissolved in 20 μL nuclease-free water.

### PCR and amplicon sequencing

Amplicons (using a volume of 10 μL in a 20 ng template) of the V3–V4 region in the bacterial 16S-rRNA-gene and amplicons of the ITS1 region in the fungal 18S-rRNA-gene were generated and Illumina-sequenced in 2018 by Eurofins Genomics (Ebersberg, Germany) (“Dataset 1”). The PCR conditions followed by library preparation and sequencing were described previously (D’Alvise et al. 2018). Primers for the V3–V4 region of the 16S-rRNA gene were 5′-TACGGGAGGCAGCAG (F) and 5′-CCAGGGTATCTAATCC (R) (Turner et al. 1999; Kisand and Wikner 2003). Primers for the fungal ITS1 region were 5′-GGAAGTAAAAGTCGTAACAAGG (F) and 5′-GCTGCGTTCTTCATCGATGC (R) (White et al. 1990). Amplicons from samples in 2019 were Illumina-sequenced by StarSEQ (Mainz, Germany) (“Dataset 2”). Primers for the V3–V4 region of the 16S-rRNA gene were 5′-CCTACGGGAGGCAGCAG (F) and 5′-GGACTACNNGGGTATCTAAT (R) (Klindworth et al. 2013). Primers for the fungal ITS1 region were 5′-CTTGGTCATTTAGAGGAAGTAA (F) and 5′-GCTGCGTTCTTCATCGATGC (R) (White et al. 1990; Gardes and Bruns 1993), an obviously modified and improved set compared to the one from Eurofins Genomics. For all primer sets, efficacies were reported to be high and evaluated by the standardized procedures of the companies.

The clean sequencing reads from the bacterial 16-rRNA-gene, as provided after quality control and trimming by the sequencing company, were analyzed on the IMNGS server platform (Lagkouvardos et al. 2016). Quality filtering showed %Q30 values of about 90% of the expected amplicon sizes. Analyses were performed without further trimming using a total abundance threshold of 0.1%, and the reads were binned on a 1% difference criterium (Edgar 2013; Lagkouvardos et al. 2016). Sequences from the fungal 18S-rRNA-gene were analyzed by StarSEQ using the QIIME2 platform. The taxonomic classification of the representative OTU sequences were controlled and refined by BLAST-searches against reference material in the NCBI database (https://blast.ncbi.nlm.nih.gov).

### Statistical analysis

Differences in the relative abundances of bacterial and fungal OTUs between locations and treatments were analyzed using an ANOVA-type generalized linear model (GLM). Since the analyzed relative abundances are bounded between zero and one, the data are generated by beta distribution (Ferrari and Cribari-Neto 2004). The model was estimated using the betareg package (Cribari-Neto and Zeileis 2010). To identify significant differences in the microbial communities between the different locations and storage conditions, a single-step multiple comparison test (Tukey’s test) was performed. The reported *p*-values were corrected for multiple testing. All statistical analyses were performed using R version 3.6.2. with a significance level of *p* = 0.05.

## Results

### Characterization of pollen diversity

The palynological analysis showed, as expected, differences in pollen diversity at each location sites. At location H, pollen composition showed a high variety with 19% *Phacelia sp*., 14% *Plantago sp*., 10% *Filipendula sp*. and *Sinapsis-type*. Location N predominantly showed pollen of 42% *Aruncus sp*. and 16% *Ligustrum sp*., followed by 8% *Tilia sp*. Location F was dominated by 42% *Acer sp*. followed by 12% pollen from *Ranunculacaea* and 10% *Aesculus sp*. (Table 1, Table S1).

**Table 1:** Classification of pollen diversity in the samples

Classification	Hohenheim 2018	Forbach 2019	Nuertingen 2019
*Aceraceae_Acer*	-	42%	-
*Boraginaceae_Phacelia*	19%	-	-
*Brassicaceae_Sinapis-T*	10%	-	-
*Malvaceae_Tilia*	8%	-	8%
*Oleaceae_Ligustrum*	-	0.2%	16%
*Plantaginaceae_Plantago*	14%	3%	5%
*Ranunculaceae*	-	12%	-
*Rosaceae_Aruncus dioicus*	-	0.4%	42%
*Rosaceae_Filipendula*	10%	-	2%
*Sapindacaea_Aesculus*	0.4%	10%	-
Others	38%	33%	28%

### Quantitative data of amplicon sequencing

The total bacterial and fungal sequences and the total number of operational taxonomic units (OTUs) from 16S (bacteria) and 18S (fungi) before and after IMNGS/QIIME2 analyzing are shown in Table 2. After filtering (> 0.1%) and binning, 38 different bacteria genera and 33 different fungal genera were obtained, and their relative abundances were calculated (Table S2 and Table S3). A cut-off of 10% was performed for statistical analysis to obtain the most abundant bacteria and fungi (Table S4 and Table S5).

**Table 2:** Sequences and OTUs within the raw data and after filtering with IMNGS and QIIME2

		Dataset 1 2018 (H)		Dataset 2 2019 (F, N)	
		Bacteria	Fungi	Bacteria	Fungi
Raw data	Sequences	164,508	1,013,947	108,399	2,893,070
	OTUs	453	1093	2215	5763
IMNGS/QIIME2	OTUs	56	131	33	1998
	Mean OTUs/sample	40.2	23.2	21.8	357.7

### Differences between Dataset 1 and Dataset 2

Given the fact that for technical reasons the samples of 2018 (Dataset 1, H) and of 2019 (Dataset 2, F and N) were processed by different companies, a stringent comparison between both is not recommended. In particular, we noticed a high deviation in the fungal composition between Dataset 1 and Dataset 2 (Table 2), because primers with different binding specifications were used by the sequencing companies for the fungal ITS1 region. Analyses from Dataset 1 provided only data from *Ascomycota*, likely as a result of using non-fungi-specific primers (see “PCR and amplicon sequencing”). Consequently, the fungal composition from Dataset 1 consists of only three different genera in the samples and probably the full spectrum of fungal diversity in the pollen samples was not revealed in Dataset 1. Therefore, results from Dataset 1 were removed and all subsequent statistical analysis was performed only with Dataset 2. For the sake of completeness, the results of Dataset 1 are listed additionally in the supplemental material (Table S6, Table S7, and Figure S3).

### Microbial diversity in fresh and stored bee pollen

**Fig. 1:**
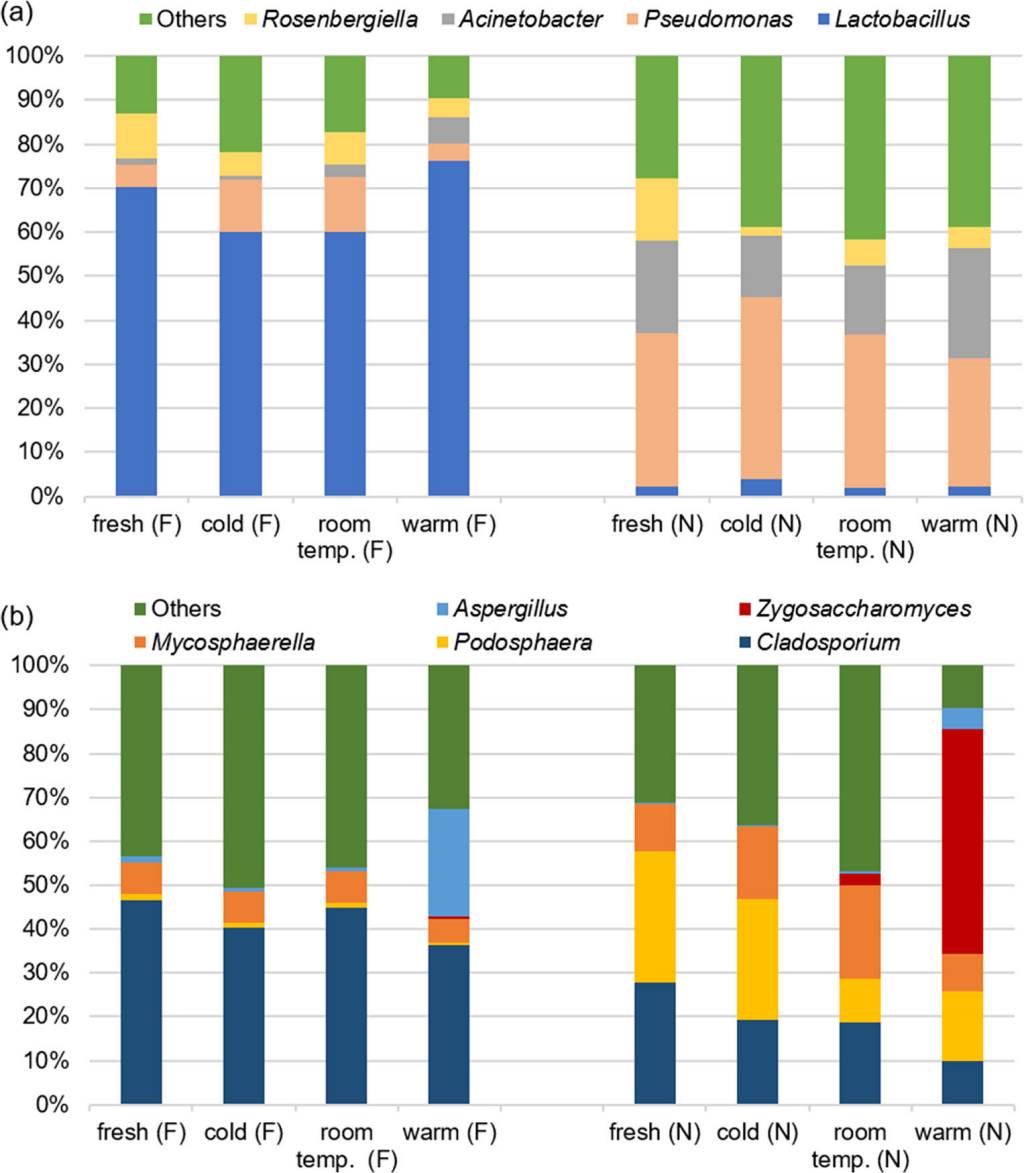
Stack bar chart, showing the composition of bacterial (**a**) and fungal (**b**) communities of Dataset 2 (F and N 2019) (filtered on minimum of 10% average) in fresh and stored bee pollen

**Fig. 2:**
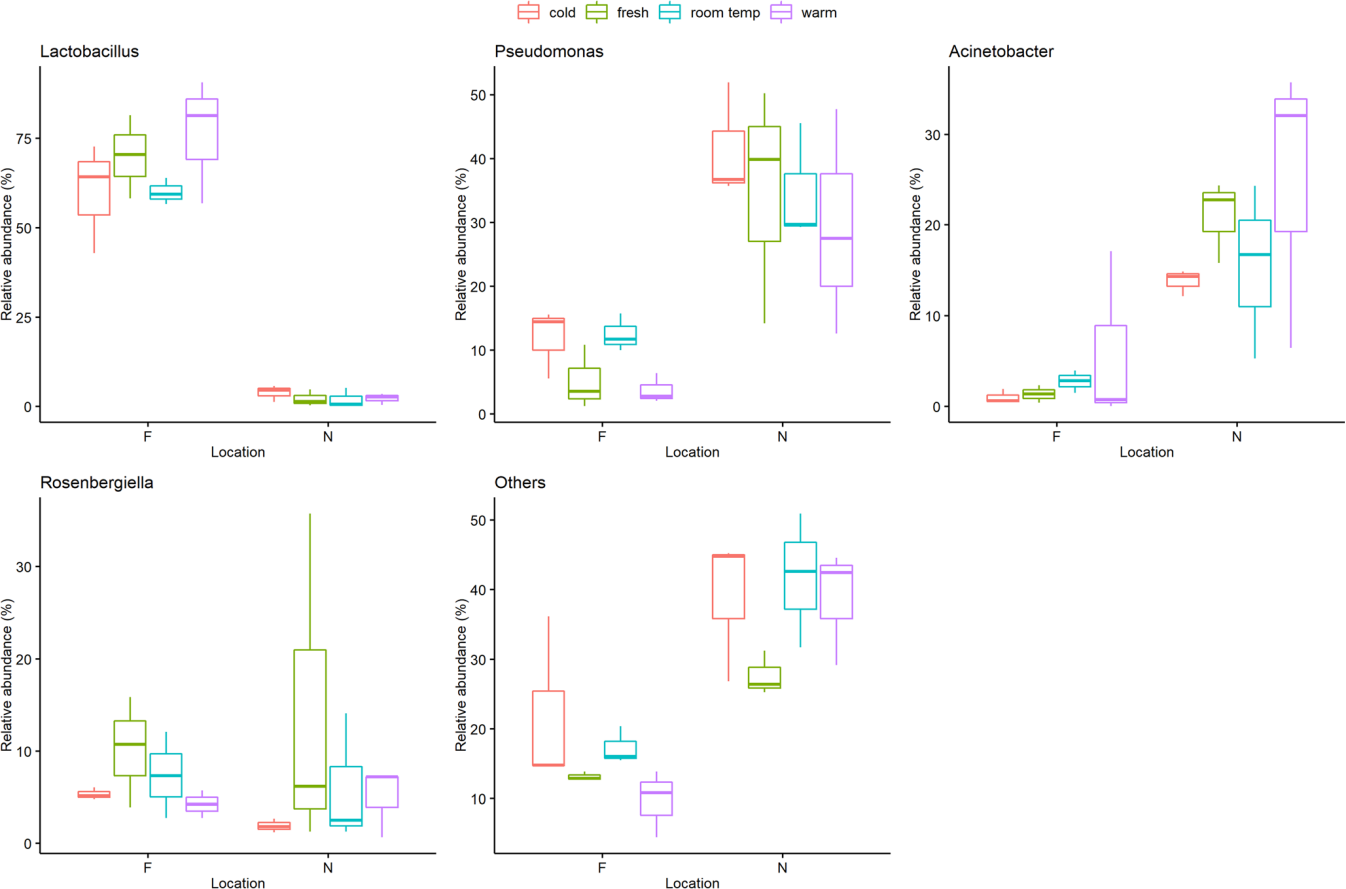
Box plots chart (created with R 3.6.2), showing that the empirical distribution of bacterial genera differs between locations and storage conditions. The estimated interquartile range is represented as a box and a line spans from the observed minimum to the observed maximum

The most abundant bacterial phyla in all samples were *Proteobacteria* (48%) and *Firmicutes* (44%), followed by *Bacteroidetes* (4%), *Actinobacteria* (3%), and *Cyanobacteria* (0.1%) in each of which between one and 26 different bacterial genera have been identified (Table 3). The main bacterial genera in Dataset 2 were *Lactobacillus* (2–76%), *Pseudomonas* (5–42%), and *Acinetobacter* (1–25%) (Fig. 1a) (Table S4). The percentage of *Acinetobacter* was higher in pollen after warm storage than in fresh pollen, while *Pseudomonas* and *Rosenbergiella* were less abundant after warm storage than in fresh pollen (Fig. 2). However, only a significant difference between storage conditions “room temperature – warm” could be found for *Lactobacillus* (GLM; *p* = 0.039; Tukey; *p* = 0.043). In contrast, the source location of the pollen samples was associated with a significant difference in relative abundance of all bacterial genera (GLM; *p* < 0.001), except *Rosenbergiella* (GLM; *p* = 0.264) (Table S6). Location F was characterized by a high proportion of *Lactobacillus* (60–76%) and low proportion of *Pseudomonas* (5–12%), while location N showed low proportion of *Lactobacillus* (2–4%) and high proportion of *Pseudomonas* (29–42%) (Fig. 1a).

**Table 3:** Bacterial phyla composition with bacterial genera calculated with total reads in all samples (Dataset 2)

Actinobacteria (3%)	*Arthrobacter*
Bacteroidetes (4%)	*Apibacter*
	*Bacteroides*
	*Chryseobacterium*
	*Epilithonimonas*
	*Flavobacterium*
	*Pedobacter*
Cyanobacteria (0.1%)	*Cyanobium*
Firmicutes (44%)	*Fusicatenibacter*
	*Lactobacillus*
	*Lactococcus*
	*Staphylococcus*
Proteobacteria (48%)	*Acinetobacter*
	*Actibacterium*
	*Arsenophonus*
	*Batronella*
	*Bradyrhizobium*
	*Carnimonas*
	*Citrobacter*
	*Duganella*
	*Erwinia*
	*Escherichia*
	*Frischella*
	*Gilliamella*
	*Gluconacetobacter*
	*Halotalea*
	*Massilia*
	*Neokomagataea*
	*Pantoea*
	*Pectobacterium*
	*Phaseolibacter*
	*Pseudomonas*
	*Rickettsia*
	*Rosenbergiella*
	*Saccharibacter*
	*Serratia*
	*Snodgrassella*
	*Sphingomonas*

**Fig. 3:**
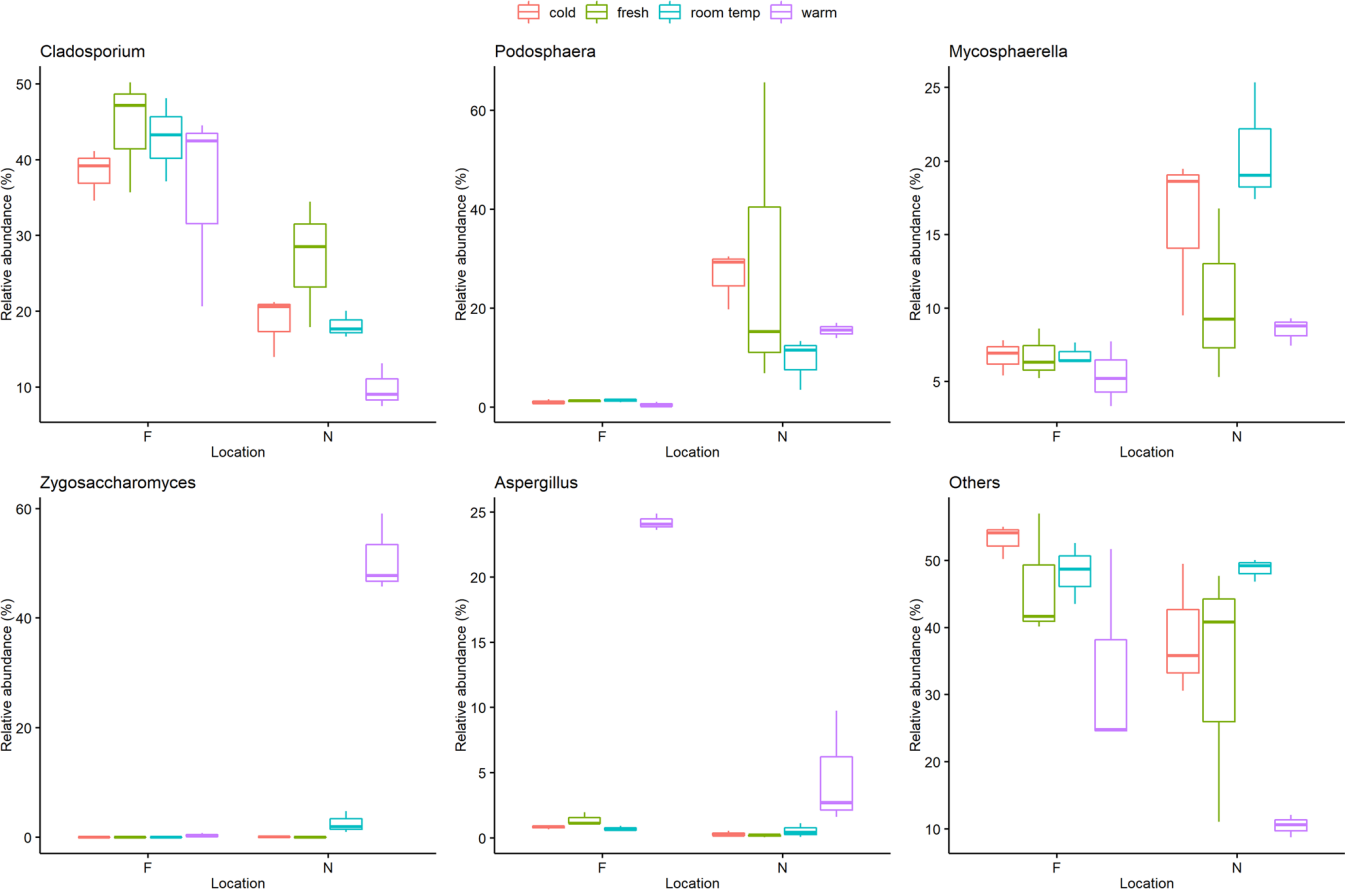
Box plots chart (created with R 3.6.2), showing that the empirical distribution of fungal genera differs between locations and storage conditions. The estimated interquartile range is represented as a box and a line spans from the observed minimum to the observed maximum

The fungal phyla from Dataset 2 are composed of 71% *Ascomycota*, 21% *Basidiomycota*, 0.15% *Motiellellomycota*, and unclassified (3.1%) (Table 4). The phylum *Ascomycota* consisted of 32 different fungal genera that were found in all samples. The main representatives of the *Ascomycota* genera in all analyzed samples were *Cladosporium* (10–45%), *Podosphaera* (1–30%), *Mycosphaerella* (5–20%), and *Zygosaccharomyces* (0–50%) (Fig. 1b) (Table S5). Significant differences between the storage conditions could be shown for fungal communities (GLM; *p* < 0.001), except for *Podosphaera* (GLM; *p* = 0.084) (Table S8). *Zygosaccharomyces* and *Aspergillus* showed significant differences between “fresh – warm,” “cold – warm,” and “room temperature – warm” conditions (Tukey; *p* < 0.001) (Table S9), while *Cladosporium* showed significant differences between “fresh – warm” and “room temperature – warm” conditions (Tukey; *p* < 0.001). The proportion of *Cladosporium* under “fresh” and “room temperature” storage conditions was significantly higher than under “warm” conditions (location N), while proportions of *Zygosaccharomyces* and *Aspergillus* significantly increased under warm storage conditions in all samples (Fig. 3). Furthermore, all locations showed also significant differences for every fungal genus (GLM; *p* < 0.001).

**Table 4:** Fungal phyla composition with fungal genera calculated with total reads in all samples (Dataset 2)

Ascomycota (71%)	*Alpinaria*
	*Alternaria*
	*Aspergillus*
	*Aureobasidium*
	*Bettsia*
	*Blumeria*
	*Cladosporium*
	*Debaryomyces*
	*Didymella*
	*Epicoccum*
	*Erysiphe*
	*Fusarium*
	*Geosmithia*
	*Leptosphaeria*
	*Metschnikowia*
	*Monilinia*
	*Monodictys*
	*Mycosphaerella*
	*Neodevriesia*
	*Neosetophoma*
	*Penicillium*
	*Periconia*
	*Phaeotheca*
	*Podosphaera*
	*Pseudoophiobolus*
	*Pyrenophora*
	*Ramularia*
	*Septoria*
	*Taphrina*
	*Tetracladium*
	*Trichomerium*
	*Zygosaccharomyces*
Basidiomycota (21%)	
Motiellellomycota (0.15%)	
Unclassified (3.1%)

## Discussion

The microorganism composition in bee pollen is affected by plant source, geographical origin, and bee keeper activities (Nogueira et al. 2012; De-Melo et al. 2016). In this study, we analyzed the botanical origin of the collected bee pollen samples. The pollen composition differed in all pollen samples; therefore, the influence of the botanical origin on the microorganism composition can be supported by the results of this study. We showed as well that the location has a significant influence on the bacterial and fungal communities associated with fresh, bee-collected pollen (GLM; *p* < 0.001). In addition, the composition and the changes of composition of microorganisms are influenced by the geographical origin of the pollen samples. However, we only included two different location sites for analysis. In order to support this statement, future studies have to be done with a larger number of samples. Major changes of microorganism composition in bee pollen occurred during storage under simulated “warm” conditions. Therefore, this study confirms our hypothesis that different storage conditions have a significant effect on the composition of microorganisms in pollen. The results demonstrate clearly that pollen has to be removed from the trap and processed immediately to prevent unwanted microorganism growing, instead of leaving pollen in the trap during hot environmental conditions. The results of this study are based on the abundances of sequences; culture-based experiments should also be followed in further studies to support the statements of this study.

Interestingly, the effect of the storage conditions on the microbial communities seems to be different for bacteria and fungi. With regard to the bacterial composition, a significant difference between storage conditions could only be identified for *Lactobacillus* (GLM; *p* = 0.039). *Lactobacillus*, represented by the species *Lactobacillus kunkeii*, is a core gut bacterium that has been found in all of the analyzed samples and could be detected in earlier studies. It is also present in corbicula pollen, “beebread,” and in floral nectar (Anderson et al. 2013; Kwong and Moran 2016). The proportion of *Lactobacillus* changes significantly between room temperature and warm conditions (Tukey; *p* = 0.043). Nevertheless, differences between storage conditions could also be determined for other bacteria. *Acinetobacter* is a bacterium needing aerobic growth condition and has been found not only in different environments, mainly in nectar of plants, but also in corbicula pollen, beebread, and in the intestinal tract of honey bees (*Apis mellifera*) (Fridman et al. 2012; Kim et al. 2014; Donkersley et al. 2018; Disayathanoowat et al. 2020). A small increase of *Acinetobacter* was observed during storage in warm conditions. Other studies also showed an increase of *Acinetobacter* in in-hive stored “bee bread” because it prefers a sugar-rich habitat (Fridman et al. 2012; Disayathanoowat et al. 2020). Two bacterial genera that are commonly found in plant material like nectar are *Pseudomonas* and *Rosenbergiella* (Fridman et al. 2012; Halpern et al. 2013). In contrast to fresh pollen, both tended to show a slight decrease during storage under warm conditions. Based on these results, it is reasonable to assume that beside changes in temperature, other factors that might influence the growth of bacteria have to be considered. Previous studies showed that the bacterial population tends to decrease under long-term storage in-hive, related to the low pH value in the hive (Anderson et al. 2014; Disayathanoowat et al. 2020). Environments with a low pH value show a high concentration of hydrogen ions, which tends to reduce bacterial growth, whereas the growth of fungi is increased (Rousk et al. 2009).

In contrast to the bacteria, we observed a consistently high influence of the storage conditions on the changes in fungal genus composition (GLM; *p* < 0.001). The fungus *Cladosporium* was the most abundant fungus in the freshly collected samples. It is ubiquitously found in indoor and outdoor environments such as air and soil (Zalar et al. 2007). In this study, we showed that the relative fraction of *Cladosporium* decreases during storage, especially under warm conditions. The fungal genera *Podosphaera* and *Mycosphaerella* can be isolated from plant environments and are both known to be plant pathogens (Crous et al. 2006; Baiswar et al. 2010; Garibaldi et al. 2012). Both were identified in freshly collected pollen as well as in stored pollen, but their proportion generally decreased during storage. However, several groups of fungi have been identified that grown more strongly under warm and humid storage conditions, especially *Zygosaccharomyces* and *Aspergillus*. Previous studies have also shown that fungal composition changes during storage of bee bread (Sinpoo et al. 2017; Detry et al. 2020; Disayathanoowat et al. 2020). Sinpoo et al. (2017) demonstrated that the high diversity of fungal communities in bee bread decreased significantly during storage time. The most dominated fungal species in corbicular pollen were *Cladosporium* and *Aspergillus*, whereas also *Zygosaccharomyce*s dominated in stored bee bread. Detry et al. (2020) also showed that the high abundance of yeast decreased within increasing storage time. However, also the yeast *Zygosaccharomyces* dominated clearly in aged bee bread. The yeast genus *Zygosaccharomyces* is very osmotolerant yeast species and has a high tolerance for different sugars. Therefore, it is known as notorious spoilage organism of sugar-rich foods and beverages such as candy, fruit juices, sugar syrups, and wine (Martorell et al. 2007; Zuehlke et al. 2013; Aneja et al. 2014; Marvig et al. 2014). *Zygosaccharomyces spp.* are also normal members of the fungal gut communities of honey bees (Yun et al. 2018); consequently, they can be transmitted from the bee saliva to the pollen. In this study, *Zygosaccharomyces spp*. was identified as the most prolifically growing microorganisms in bee pollen stored under warm temperatures. Therefore, it seems likely that *Zygosaccharomyces* can spoil bee pollen in warm and humid storage conditions, as they producing ethanol or carbon dioxide from sugar. Pollen, used as food supplement for human, should not contain any spoilage yeast, otherwise the aroma and sensory can be influenced by fermentation (Sperber and Doyle 2009). However, since the yeast is probably already being transferred from the bees to the pollen, it is very important to prevent their reproduction and growth. The growth of such yeasts can be prevented by freezing or cooling. In our study, we did not observe any increase in the *Zygosaccharomyces* proportion at 4 °C. Other studies showed as well that *Zygosaccharomyces* growth is clearly reduced even at 8 °C (Marvig et al. 2014). Also, an increase in the *Aspergillus* proportion in the samples stored under warm conditions has to be observed with regard to human nutrition. *Aspergillus* is extremely halo- and osmotolerant (Stratford et al. 2019) and the genus contains a number of highly mycotoxigenic species (González et al. 2005). Some species of this fungal genus have been identified as pathogens in insects, animals, and humans (Foley et al. 2014; Dagenais and Keller 2009). In food, *Aspergillus spp.* can spoil as visible growth of black mold, discoloration, or in producing mycotoxins. The effects of mycotoxins on human health are complex and can cause cancerogenic effects or central nervous system damage (Sperber and Doyle 2009). Since the growth of *Aspergillu*s can cause major effects on human health, it is particularly important to prevent the growing in pollen samples. Another study reported findings of mycotoxin-producing *Aspergillus spp*. in “ready-to-eat” pollen samples from Spain (González et al. 2005). The fungal contamination in the study from González et al. indicated that the post-harvest pollen processed negatively impacted pollen quality. Incorrect storage or drying conditions as well as non-daily harvest were pointed out as reasons for the contamination.

In conclusion, pollen stored under warm conditions showed the clearest changes in fungal composition, compared to the freshly collected pollen (Tukey; *p* < 0.001). Growth of fungi from the genera *Zygosaccharomyces* or *Aspergillus* was likely the cause of spoilage. Therefore, during processing of freshly harvested bee pollen, it is important to prevent growth of these spoilage microorganisms. This is most conveniently achieved by harvesting daily, followed by processing the pollen directly to refrigeration or, even better, freezing.

## Supplementary information

ESM 1 (PDF 598 kb).
