# Supplementary material for: Changes of microorganism composition in fresh and stored bee pollen from Southern Germany
Source: Environ Sci Pollut Res Int. 2021 Apr 23;28(34):47251–61. doi: 10.1007/s11356-021-13932-4 (PMC8692275; doi:10.1007/s11356-021-13932-4)

Supporting information for:

Changes of microorganism composition in fresh and stored bee pollen from Southern Germany

Carolin Friedle [1,2*] · Paul D'Alvise [2] · Karsten Schweikert [3] · Klaus Wallner [1] · Martin Hasselmann [2]

[1]University of Hohenheim, Apicultural State Institute, Stuttgart, Germany

[2]University of Hohenheim, Institute of Animal Science, Department of Livestock Population Genomics, Stuttgart, Germany

[3]University of Hohenheim, Core Facility Hohenheim and Institute of Economics, Stuttgart, Germany

*carolin_friedle@uni-hohenheim.de

Tables S2-S5 with coding:

First: F = fresh; C = cold; RT = room temperature; W = warm

Second: Numbers 1-3 for replicates

Third: H = Hohenheim; F = Forbach; N = Nuertingen

Table S1 Detailed palynological analysis in all samples

classification	Hohenheim 2018	Forbach 2019	Nuertingen 2019
Aceraceae_Acer		208	
Sapindacaea_Aesculus	2	49	
Apiaceae			4
Rosaceae_Aruncus dioicus		2	209
Asteraceae_Bidens-T	2		
Brassicaceae_Brassica-T	38		
Asteraceae_Carduus	22		
Cyperaceae_Carex		8	
Fagaceae_Castanea sativa	4		
Chenopodiaceae	2		
Cornaceae_Cornus-T		3	
Boraginaceae_Echium	8		
Fabaceae		34	
Rosaceae_Filipendula	52		10
Rosaceae_Fragaria			2
Apiaceae_Heracleum		12	
Cannabaceae_Humulus	2		
Clusiaceae_Hypericum	1	4	3
Balsaminaceae_Impatiens	1		
Lamiaceae_Lamium-T		3	14
Oleaceae_Ligustrum		1	78
Fabaceae_Melilotus	1	2	
Papaveraceae		1	4
Boraginaceae_Phacelia	96		
Pinaceae_Pinoideae		31	
Plantaginaceae_Plantago	72	17	24
Poaceae	8		2
Rosaceae_Potentilla			44
Ranunculaceae		59	
Orobanchaceae_Rhinanthus			16
Anacardiaceae_Rhus typhina			2
Rosaceae_Rosa			41
Rosaceae_Rubus-T	3	6	1
Polygonaceae_Rumex	2		3
Rosaceae_Sanguisorba minor		16	1
Cyperaceae_Scirpus		35	
Crassulaceae_Sedum	44		
Brassicaceae_Sinapis-T	51		
Asteraceae_Taraxacum-T	27		
Malvaceae_Tilia	39		39
Fabaceae_Trifolium repens	18	1	3
Adoxaceae_Viburnum		8	
Poaceae_Zea mays	5		

Table S2 Calculated relative abundance of all detected bacteria in the samples

F1H	F2H	F3H	C1H	C2H	C3H	RT1H	RT2H	W1H	W2H	W3H	taxonomy
0.4335	0.6673	0.8104	0.5924	0.7750	0.6638	0.8669	0.9062	0.5248	0.0748	0.5476	Bacteria;Firmicutes;Bacilli;Lactobacillales;Lactobacillaceae;Lactobacillus;
0.1430	0.0173	0.0255	0.0070	0.0255	0.0864	0.0149	0.0067	0.0011	0	0.0174	Bacteria;Proteobacteria;Gammaproteobacteria;Pseudomonadales;Pseudomonadaceae;Pseudomonas;
0.0256	0.0505	0.0043	0.2206	0.0079	0.0834	0.0022	0.0033	0.0246	0.0200	0.2077	Bacteria;Proteobacteria;Gammaproteobacteria;Pseudomonadales;Moraxellaceae;Acinetobacter;
0.1204	0.0038	0.0084	0.0041	0.0025	0.0064	0.0976	0.0012	0	0	0.0321	Bacteria;Proteobacteria;Gammaproteobacteria;Enterobacteriales;Enterobacteriaceae;Rosenbergiella;
0.0962	0.0874	0.0167	0.0070	0.0059	0.0787	0.0016	0.0014	0.0089	0	0.0056	Bacteria;Proteobacteria;Gammaproteobacteria;Enterobacteriales;Erwiniaceae;Pantoea
0.0124	0.0117	0.0053	0.0014	0.0032	0.0045	0.0001	0.0006	0.3230	0.7537	0.0683	Bacteria;Actinobacteria;Actinobacteria;Actinomycetales;Micrococcaceae;Arthrobacter;
0.0205	0.0085	0.0054	0.0106	0.0121	0.0163	0.0004	0.0095	0.0051	0	0.0137	Bacteria;Proteobacteria;Gammaproteobacteria;Enterobacteriales;Enterobacteriaceae;Erwinia;
0	0	0	0	0	0	0	0	0	0	0	Bacteria;Bacteroidetes;Flavobacteriia;Flavobacteriales;Flavobacteriaceae;Epilithonimonas;
0	0	0	0	0	0	0	0	0	0	0	Bacteria;Proteobacteria;Gammaproteobacteria;Enterobacteriales;Erwiniaceae;Phaseolibacter;
0.0106	0.0030	0.0828	0.0004	0.0015	0.0026	0.0002	0.0007	0	0.0002	0	Bacteria;Proteobacteria;Gammaproteobacteria;Enterobacteriales;Enterobacteriaceae;Serratia;
0.0538	0.0566	0.0148	0.0695	0.0862	0.0323	0.0024	0.0297	0.0212	0.0001	0.0249	Bacteria;Proteobacteria;Gammaproteobacteria;Enterobacteriales;Enterobacteriaceae;Arsenophonus;
0.0118	0.0357	0.0068	0.0134	0.0211	0.0103	0.0086	0.0127	0.0080	0	0.0235	Bacteria;Proteobacteria;Alphaproteobacteria;Rhodospirillales;Acetobacteraceae;Saccharibacter;
0.0097	0.0130	0.0019	0.0236	0.0059	0.0034	0.0017	0.0096	0.0246	0.0215	0.0194	Bacteria;Proteobacteria;Alphaproteobacteria;Sphingomonadales;Sphingomonadaceae;Sphingomonas;
0.0005	0	0	0.0006	0.0002	0.0001	0.0001	0.0038	0	0	0	Bacteria;Bacteroidetes;Flavobacteriia;Flavobacteriales;Flavobacteriaceae;Chryseobacterium;
0.0063	0.0034	0.0012	0.0084	0.0027	0.0039	0.0007	0.0033	0.0159	0.0210	0.0132	Bacteria;Proteobacteria;Betaproteobacteria;Burkholderiales;Oxalobacteraceae;Massilia;
0.0004	0.0035	0.0006	0.0015	0.0097	0.0005	0.0000	0.0032	0	0	0.0011	Bacteria;Proteobacteria;Alphaproteobacteria;Rhodospirillales;Acetobacteraceae;Gluconacetobacter;
0	0	0	0	0	0	0	0	0	0	0	Bacteria;Proteobacteria;Betaproteobacteria;Burkholderiales;Oxalobacteraceae;Duganella;
0.0018	0.0016	0.0002	0.0018	0.0009	0.0001	0.0001	0.0005	0.0019	0.0101	0.0017	Bacteria;Bacteroidetes;Sphingobacteriia;Sphingobacteriales;Sphingobacteriaceae;Pedobacter;
0.0022	0.0085	0.0044	0.0081	0.0095	0.0043	0.0006	0.0013	0	0.0096	0.0064	Bacteria;Proteobacteria;Gammaproteobacteria;Orbales;Orbaceae;Gilliamella;
0	0	0	0	0	0	0	0	0	0	0	Bacteria;Cyanobacteria;Cyanobacteria;Synechococcales; Synechococcaceae; Cyanobium
0	0	0	0	0	0	0	0	0.0114	0.0362	0	Bacteria;Firmicutes;Clostridia;Clostridiales;Lachnospiraceae;Fusicatenibacter;
0.0017	0.0085	0.0008	0.0056	0.0032	0.0006	0.0005	0.0020	0.0234	0	0	Bacteria;Proteobacteria;Gammaproteobacteria;Oceanospirillales;Halomonadaceae;Carnimonas;
0.0003	0.0026	0.0002	0.0027	0.0046	0.0006	0.0001	0.0010	0.0060	0.0154	0.0079	Bacteria;Proteobacteria;Gammaproteobacteria;Enterobacteriales;Enterobacteriaceae;Escherichia/Shigella;
0.0170	0.0081	0.0029	0.0014	0.0006	0.0006	0.0006	0.0004	0	0	0	Bacteria;Proteobacteria;Gammaproteobacteria;Enterobacteriales;Enterobacteriaceae;Citrobacter;
0.0127	0	0.0004	0.0005	0.0006	0.0001	0.0000	0.0005	0	0.0139	0.0007	Bacteria;Firmicutes;Bacilli;Lactobacillales;Streptococcaceae;Lactococcus;
0	0	0	0	0	0	0	0	0	0	0	Bacteria;Proteobacteria;Alphaproteobacteria;Rhizobiales;Bradyrhizobiaceae;Bradyrhizobium;

0	0	0	0	0	0	0	0	0	0.0182	0.0011	Bacteria;Bacteroidetes;Bacteroidia;Bacteroidales;Bacteroidaceae;Bacteroides;
0.0160	0.0004	0.0012	0.0002	0.0005	0.0002	0.0001	0.0003	0	0	0	Bacteria;Proteobacteria;Alphaproteobacteria;Rickettsiales;Rickettsiaceae;Rickettsia;
0	0	0	0	0	0	0	0	0	0	0	Bacteria;Proteobacteria;Alphaproteobacteria;Rhodobacterales;Rhodobacteraceae;Actibacterium
0.0012	0.0023	0.0019	0.0043	0.0039	0.0003	0.0000	0.0006	0	0	0.0010	Bacteria;Proteobacteria;Betaproteobacteria;Neisseriales;Neisseriaceae;Snodgrassella;
0	0	0	0	0	0	0	0	0	0	0	Bacteria;Proteobacteria;Gammaproteobacteria;Enterobacteriales;Enterobacteriaceae;Pectobacterium
0.0008	0.0027	0.0002	0.0036	0.0007	0.0001	0.0001	0.0003	0	0.0001	0.0035	Bacteria;Proteobacteria;Alphaproteobacteria;Rhodospirillales;Acetobacteraceae;Neokomagataea;
0.0011	0.0012	0.0013	0.0016	0.0033	0.0003	0.0002	0.0010	0	0	0.0015	Bacteria;Proteobacteria;Gammaproteobacteria;Orbales;Orbaceae;Frischella;
0.0004	0.0015	0.0024	0.0005	0.0050	0.0002	0.0001	0.0004	0	0	0.0005	Bacteria;Proteobacteria;Alphaproteobacteria;Rhizobiales;Bartonellaceae;Batronella;
0	0.0003	0	0.0001	0.0075	0	0.0000	0.0000	0	0	0	Bacteria;Bacteroidetes;Flavobacteriia;Flavobacteriales;Flavobacteriaceae;Apibacter;
0.0002	0.0007	0.0000	0.0002	0.0002	0.0001	0.0000	0.0001	0	0.0052	0	Bacteria;Firmicutes;Bacilli;Bacillales;Staphylococcaceae;Staphylococcus;
0	0	0	0.0051	0	0.0001	0	0	0	0	0.0012	Bacteria;Proteobacteria;Gammaproteobacteria;Oceanospirillales;Halomonadaceae;Halotalea;
0	0	0	0.0039	0	0	0.0000	0.0000	0	0	0	Bacteria;Bacteroidetes;Flavobacteriia;Flavobacteriales;Flavobacteriaceae;Flavobacterium;

F1F	F2F	F3F	C1F	C2F	C3F	RT1F	RT2F	RT3F	W1F	W2F	W3F	taxonomy
0.5824	0.8154	0.7050	0.7270	0.4291	0.6423	0.5668	0.5939	0.6394	0.9061	0.5689	0.8142	Bacteria;Firmicutes;Bacilli;Lactobacillales;Lactobacillaceae;Lactobacillus;
0.1086	0.0126	0.0356	0.0557	0.1557	0.1447	0.1173	0.1578	0.1004	0.0211	0.0641	0.0277	Bacteria;Proteobacteria;Gammaproteobacteria;Pseudomonadales;Pseudomonadaceae;Pseudomonas;
0.0233	0.0041	0.0137	0.0192	0.0062	0.0046	0.0398	0.0150	0.0285	0.0005	0.1711	0.0076	Bacteria;Proteobacteria;Gammaproteobacteria;Pseudomonadales;Moraxellaceae;Acinetobacter;
0.1586	0.0390	0.1071	0.0515	0.0477	0.0603	0.1209	0.0730	0.0275	0.0275	0.0572	0.0423	Bacteria;Proteobacteria;Gammaproteobacteria;Enterobacteriales;Enterobacteriaceae;Rosenbergiella;
0.0139	0.0043	0.0072	0.0461	0.0092	0.0252	0.0072	0.0189	0.0017	0.0055	0.0016	0.0174	Bacteria;Proteobacteria;Gammaproteobacteria;Enterobacteriales;Erwiniaceae;Pantoea
0	0	0	0	0	0	0	0	0	0	0	0	Bacteria;Actinobacteria;Actinobacteria;Actinomycetales;Micrococcaceae;Arthrobacter;
0.0080	0.0046	0.0436	0.0050	0.0101	0.0226	0.0202	0.0183	0.0062	0.0005	0.0276	0.0024	Bacteria;Proteobacteria;Gammaproteobacteria;Enterobacteriales;Enterobacteriaceae;Erwinia;
0	0	0	0	0.0003	0.0003	0	0	0.0002	0	0	0.0003	Bacteria;Bacteroidetes;Flavobacteriia;Flavobacteriales;Flavobacteriaceae;Epilithonimonas;
0.0417	0.0623	0.0432	0.0398	0.2524	0.0529	0.0583	0.0469	0.0449	0.0033	0.0328	0.0484	Bacteria;Proteobacteria;Gammaproteobacteria;Enterobacteriales;Erwiniaceae;Phaseolibacter;
0.0242	0.0151	0.0104	0.0231	0.0359	0.0037	0.0101	0.0111	0.1169	0.0008	0.0126	0.0049	Bacteria;Proteobacteria;Gammaproteobacteria;Enterobacteriales;Enterobacteriaceae;Serratia;
0	0.0006	0.0006	0	0	0	0	0	0	0	0.0004	0	Bacteria;Proteobacteria;Gammaproteobacteria;Enterobacteriales;Enterobacteriaceae;Arsenophonus;
0.0116	0.0351	0.0259	0.0195	0.0096	0.0203	0.0081	0.0274	0.0212	0.0168	0.0126	0.0183	Bacteria;Proteobacteria;Alphaproteobacteria;Rhodospirillales;Acetobacteraceae;Saccharibacter;
0.0057	0.0007	0.0012	0.0021	0.0039	0.0057	0.0046	0.0124	0.0031	0.0025	0.0191	0.0058	Bacteria;Proteobacteria;Alphaproteobacteria;Sphingomonadales;Sphingomonadaceae;Sphingomonas;
0.0001	0	0	0	0	0	0	0.0007	0	0	0.0004	0.0003	Bacteria;Bacteroidetes;Flavobacteriia;Flavobacteriales;Flavobacteriaceae;Chryseobacterium;
0.0105	0.0041	0.0050	0.0067	0.0140	0.0129	0.0117	0.0176	0.0077	0.0146	0.0195	0.0058	Bacteria;Proteobacteria;Betaproteobacteria;Burkholderiales;Oxalobacteraceae;Massilia;
0	0.0011	0	0	0.0002	0	0	0	0	0.0005	0.0004	0	Bacteria;Proteobacteria;Alphaproteobacteria;Rhodospirillales;Acetobacteraceae;Gluconacetobacter;
0.0050	0.0004	0.0001	0.0011	0.0017	0.0017	0.0111	0.0065	0.0013	0	0.0032	0.0006	Bacteria;Proteobacteria;Betaproteobacteria;Burkholderiales;Oxalobacteraceae;Duganella;
0.0003	0.0002	0	0.0007	0	0.0026	0.0238	0.0007	0.0010	0.0003	0.0004	0	Bacteria;Bacteroidetes;Sphingobacteriia;Sphingobacteriales;Sphingobacteriaceae;Pedobacter;
0.0001	0.0002	0.0001	0	0.0226	0.0003	0	0	0	0	0	0	Bacteria;Proteobacteria;Gammaproteobacteria;Orbales;Orbaceae;Gilliamella;
0.0060	0.0002	0.0013	0.0021	0	0	0	0	0	0	0.0077	0.0040	Bacteria;Cyanobacteria;Cyanobacteria;Synechococcales; Synechococcaceae; Cyanobium
0	0	0	0	0	0	0	0	0	0	0	0	Bacteria;Firmicutes;Clostridia;Clostridiales;Lachnospiraceae;Fusicatenibacter;
0	0	0	0	0	0	0	0	0	0	0	0	Bacteria;Proteobacteria;Gammaproteobacteria;Oceanospirillales;Halomonadaceae;Carnimonas;
0	0	0	0	0	0	0	0	0	0	0	0	Bacteria;Proteobacteria;Gammaproteobacteria;Enterobacteriales;Enterobacteriaceae;Escherichia/Shigella;
0	0	0	0	0	0	0	0	0	0	0	0	Bacteria;Proteobacteria;Gammaproteobacteria;Enterobacteriales;Enterobacteriaceae;Citrobacter;
0	0	0	0	0	0	0	0	0	0	0	0	Bacteria;Firmicutes;Bacilli;Lactobacillales;Streptococcaceae;Lactococcus;
0	0	0	0	0	0	0	0	0	0	0	0	Bacteria;Proteobacteria;Alphaproteobacteria;Rhizobiales;Bradyrhizobiaceae;Bradyrhizobium;
0	0	0	0	0	0	0	0	0	0	0	0	Bacteria;Bacteroidetes;Bacteroidia;Bacteroidales;Bacteroidaceae;Bacteroides;
0	0	0	0	0	0	0	0	0	0	0	0	Bacteria;Proteobacteria;Alphaproteobacteria;Rickettsiales;Rickettsiaceae;Rickettsia;

0	0	0	0	0	0	0	0	0	0	0	0	Bacteria;Proteobacteria;Alphaproteobacteria;Rhodobacterales;Rhodobacteraceae;Actibacterium
0	0	0	0	0	0	0	0	0	0	0	0	Bacteria;Proteobacteria;Betaproteobacteria;Neisseriales;Neisseriaceae;Snodgrassella;
0	0	0	0.0004	0.0014	0	0	0	0	0	0.0004	0	Bacteria;Proteobacteria;Gammaproteobacteria;Enterobacteriales;Enterobacteriaceae;Pectobacterium
0	0	0	0	0	0	0	0	0	0	0	0	Bacteria;Proteobacteria;Alphaproteobacteria;Rhodospirillales;Acetobacteraceae;Neokomagataea;
0	0	0	0	0	0	0	0	0	0	0	0	Bacteria;Proteobacteria;Gammaproteobacteria;Orbales;Orbaceae;Frischella;
0	0	0	0	0	0	0	0	0	0	0	0	Bacteria;Proteobacteria;Alphaproteobacteria;Rhizobiales;Bartonellaceae;Batronella;
0	0	0	0	0	0	0	0	0	0	0	0	Bacteria;Bacteroidetes;Flavobacteriia;Flavobacteriales;Flavobacteriaceae;Apibacter;
0	0	0	0	0	0	0	0	0	0	0	0	Bacteria;Firmicutes;Bacilli;Bacillales;Staphylococcaceae;Staphylococcus;
0	0	0	0	0	0	0	0	0	0	0	0	Bacteria;Proteobacteria;Gammaproteobacteria;Oceanospirillales;Halomonadaceae;Halotalea;
0	0	0	0	0	0	0	0	0	0	0	0	Bacteria;Bacteroidetes;Flavobacteriia;Flavobacteriales;Flavobacteriaceae;Flavobacterium;

F1N	F2N	F3N	C1N	C2N	C3N	RT1N	RT2N	RT3N	W1N	W2N	W3N	taxonomy
0.0479	0.0035	0.0143	0.0459	0.0570	0.0135	0.0015	0.0062	0.0524	0.0348	0.0273	0.0045	Bacteria;Firmicutes;Bacilli;Lactobacillales;Lactobacillaceae;Lactobacillus;
0.3984	0.1424	0.5023	0.3574	0.5189	0.3670	0.2967	0.2924	0.4553	0.1264	0.4771	0.2746	Bacteria;Proteobacteria;Gammaproteobacteria;Pseudomonadales;Pseudomonadaceae;Pseudomonas;
0.2276	0.2436	0.1581	0.1220	0.1436	0.1489	0.2432	0.1671	0.0531	0.3207	0.0647	0.3570	Bacteria;Proteobacteria;Gammaproteobacteria;Pseudomonadales;Moraxellaceae;Acinetobacter;
0.0617	0.3576	0.0128	0.0267	0.0121	0.0183	0.1409	0.0250	0.0128	0.0723	0.0066	0.0719	Bacteria;Proteobacteria;Gammaproteobacteria;Enterobacteriales;Enterobacteriaceae;Rosenbergiella;
0.0806	0.0144	0.0951	0.0343	0.0410	0.0448	0.0661	0.0399	0.0838	0.1250	0.0755	0.0419	Bacteria;Proteobacteria;Gammaproteobacteria;Enterobacteriales;Erwiniaceae;Pantoea
0	0	0	0	0	0	0	0	0	0	0	0	Bacteria;Actinobacteria;Actinobacteria;Actinomycetales;Micrococcaceae;Arthrobacter;
0.0369	0.0199	0.0373	0.0918	0.0236	0.0109	0.0425	0.0325	0.1743	0.2005	0.0360	0.0374	Bacteria;Proteobacteria;Gammaproteobacteria;Enterobacteriales;Enterobacteriaceae;Erwinia;
0.0447	0.0756	0.0543	0.1232	0.0694	0.0446	0.0083	0.2012	0.0711	0.0182	0.1237	0.0746	Bacteria;Bacteroidetes;Flavobacteriia;Flavobacteriales;Flavobacteriaceae;Epilithonimonas;
0.0016	0	0	0.0029	0.0026	0	0.0001	0.0018	0	0.0016	0.0007	0.0009	Bacteria;Proteobacteria;Gammaproteobacteria;Enterobacteriales;Erwiniaceae;Phaseolibacter;
0.0152	0.0242	0.0126	0.0279	0.0644	0.0212	0.0250	0.0149	0.0092	0.0104	0.0273	0.0353	Bacteria;Proteobacteria;Gammaproteobacteria;Enterobacteriales;Enterobacteriaceae;Serratia;
0.0010	0.0002	0	0.0174	0.0086	0.1032	0.0005	0.0010	0.0010	0.0088	0.0388	0.0003	Bacteria;Proteobacteria;Gammaproteobacteria;Enterobacteriales;Enterobacteriaceae;Arsenophonus;
0.0131	0.0037	0.0049	0.0401	0.0132	0.0065	0.0041	0.0087	0.0074	0.0158	0.0119	0.0054	Bacteria;Proteobacteria;Alphaproteobacteria;Rhodospirillales;Acetobacteraceae;Saccharibacter;
0.0183	0.0067	0.0259	0.0540	0.0067	0.0102	0.0054	0.0238	0.0064	0.0075	0.0147	0.0150	Bacteria;Proteobacteria;Alphaproteobacteria;Sphingomonadales;Sphingomonadaceae;Sphingomonas;
0.0204	0.0680	0.0151	0.0267	0.0183	0.0216	0.0017	0.1237	0.0213	0.0080	0.0266	0.0249	Bacteria;Bacteroidetes;Flavobacteriia;Flavobacteriales;Flavobacteriaceae;Chryseobacterium;
0.0110	0.0088	0.0116	0.0139	0.0082	0.0220	0.0134	0.0050	0.0154	0.0147	0.0115	0.0174	Bacteria;Proteobacteria;Betaproteobacteria;Burkholderiales;Oxalobacteraceae;Massilia;
0.0031	0.0009	0.0012	0.0041	0.0060	0.0891	0.1298	0.0017	0.0173	0.0110	0.0021	0.0018	Bacteria;Proteobacteria;Alphaproteobacteria;Rhodospirillales;Acetobacteraceae;Gluconacetobacter;
0.0139	0.0098	0.0146	0.0023	0.0018	0.0698	0.0062	0.0189	0.0104	0.0118	0.0220	0.0279	Bacteria;Proteobacteria;Betaproteobacteria;Burkholderiales;Oxalobacteraceae;Duganella;
0.0044	0.0204	0.0086	0.0093	0.0044	0.0077	0.0017	0.0357	0.0087	0.0016	0.0091	0.0084	Bacteria;Bacteroidetes;Sphingobacteriia;Sphingobacteriales;Sphingobacteriaceae;Pedobacter;
0.0003	0	0	0	0	0	0	0	0	0	0	0	Bacteria;Proteobacteria;Gammaproteobacteria;Orbales;Orbaceae;Gilliamella;
0	0.0002	0.0143	0	0.0001	0.0008	0	0.0003	0	0.0107	0	0.0009	Bacteria;Cyanobacteria;Cyanobacteria;Synechococcales; Synechococcaceae; Cyanobium
0	0	0	0	0	0	0	0	0	0	0	0	Bacteria;Firmicutes;Clostridia;Clostridiales;Lachnospiraceae;Fusicatenibacter;
0	0	0	0	0	0	0	0	0	0	0	0	Bacteria;Proteobacteria;Gammaproteobacteria;Oceanospirillales;Halomonadaceae;Carnimonas;
0	0	0	0	0	0	0	0	0	0	0	0	Bacteria;Proteobacteria;Gammaproteobacteria;Enterobacteriales;Enterobacteriaceae;Escherichia/Shigella;
0	0	0	0	0	0	0	0	0	0	0	0	Bacteria;Proteobacteria;Gammaproteobacteria;Enterobacteriales;Enterobacteriaceae;Citrobacter;
0	0	0	0	0	0	0	0	0	0	0	0	Bacteria;Firmicutes;Bacilli;Lactobacillales;Streptococcaceae;Lactococcus;
0	0	0	0	0	0	0	0	0	0	0.0245	0	Bacteria;Proteobacteria;Alphaproteobacteria;Rhizobiales;Bradyrhizobiaceae;Bradyrhizobium;
0	0	0	0	0	0	0	0	0	0	0	0	Bacteria;Bacteroidetes;Bacteroidia;Bacteroidales;Bacteroidaceae;Bacteroides;
0	0	0	0	0	0	0	0	0	0	0	0	Bacteria;Proteobacteria;Alphaproteobacteria;Rickettsiales;Rickettsiaceae;Rickettsia;

0	0	0.0168	0	0	0	0	0	0	0	0	0	Bacteria;Proteobacteria;Alphaproteobacteria;Rhodobacterales;Rhodobacteraceae;Actibacterium
0	0	0	0	0	0	0	0	0	0	0	0	Bacteria;Proteobacteria;Betaproteobacteria;Neisseriales;Neisseriaceae;Snodgrassella;
0	0	0	0	0	0	0.0129	0	0	0	0	0	Bacteria;Proteobacteria;Gammaproteobacteria;Enterobacteriales;Enterobacteriaceae;Pectobacterium
0	0	0	0	0	0	0	0	0	0	0	0	Bacteria;Proteobacteria;Alphaproteobacteria;Rhodospirillales;Acetobacteraceae;Neokomagataea;
0	0	0	0	0	0	0	0	0	0	0	0	Bacteria;Proteobacteria;Gammaproteobacteria;Orbales;Orbaceae;Frischella;
0	0	0	0	0	0	0	0	0	0	0	0	Bacteria;Proteobacteria;Alphaproteobacteria;Rhizobiales;Bartonellaceae;Batronella;
0	0	0	0	0	0	0	0	0	0	0	0	Bacteria;Bacteroidetes;Flavobacteriia;Flavobacteriales;Flavobacteriaceae;Apibacter;
0	0	0	0	0	0	0	0	0	0	0	0	Bacteria;Firmicutes;Bacilli;Bacillales;Staphylococcaceae;Staphylococcus;
0	0	0	0	0	0	0	0	0	0	0	0	Bacteria;Proteobacteria;Gammaproteobacteria;Oceanospirillales;Halomonadaceae;Halotalea;
0	0	0	0	0	0	0	0	0	0	0	0	Bacteria;Bacteroidetes;Flavobacteriia;Flavobacteriales;Flavobacteriaceae;Flavobacterium;

Table S3 Calculated relative abundance of all detected fungi in the samples

F1H	F2H	F3H	C1H	C2H	C3H	RT1H	RT2H	RT3H	W1H	W2H	W3H	taxonomy
1	1	0.8186	1	0.5	1	1	0.4711	0.7432	0.1104	0.1276	0.5791	Fungi; Ascomycota; Dothideomycetes; Capnodiales; Cladosporiaceae; Cladosporium
0	0	0	0	0	0	0	0.3772	0	0	0	0	Fungi; Ascomycota; Leotiomycetes; Erysiphales; Erysiphaceae; Podosphaera
0	0	0	0	0	0	0	0	0	0	0	0	Fungi; Ascomycota; Dothideomycetes; Capnodiales; Mycosphaerellaceae; Mycosphaerella
0	0	0	0	0	0	0	0.1517	0	0.8525	0.6914	0.3141	Fungi; Ascomycota; Saccharomycetes; Saccharomycetales; Saccharomycetaceae; Zygosaccharomyces
0	0	0	0	0	0	0	0	0	0	0	0	Fungi; Ascomycota; Eurotiomycetes; Eurotiales; Aspergillaceae; Aspergillus
0	0	0	0	0	0	0	0	0	0	0	0	Fungi; Ascomycota; Eurotiomycetes; Eurotiales; Aspergillaceae; Penicillium
0	0	0	0	0	0	0	0	0	0	0	0	Fungi; Ascomycota; Saccharomycetes; Saccharomycetales; Metschnikowiaceae; Metschnikowia
0	0	0	0	0	0	0	0	0	0	0	0	Fungi; Ascomycota; Sordariomycetes; Hypocreales; Nectriaceae; Fusarium
0	0	0	0	0	0	0	0	0	0	0	0	Fungi; Ascomycota; Dothideomycetes; Capnodiales; Mycosphaerellaceae; Ramularia
0	0	0	0	0	0	0	0	0	0	0	0	Fungi; Ascomycota; Taphrinomycetes; Taphrinales; Taphrinaceae; Taphrina
0	0	0	0	0	0	0	0	0	0	0	0	Fungi; Ascomycota; Dothideomycetes; Pleosporales; Pleosporaceae; Alternaria
0	0	0	0	0	0	0	0	0	0	0	0	Fungi; Ascomycota; Dothideomycetes; Pleosporales; Didymellaceae; Didymella
0	0	0	0	0	0	0	0	0	0	0	0	Fungi; Ascomycota; Leotiomycetes; Thelebolales; Pseudeurotiaceae; Bettsia
0	0	0	0	0	0	0	0	0	0	0	0	Fungi; Ascomycota; Leotiomycetes; Helotiales; Sclerotiniaceae; Monilinia
0	0	0	0	0	0	0	0	0	0	0	0	Fungi; Ascomycota; Dothideomycetes; Dothideales; Aureobasidiaceae; Aureobasidium
0	0	0	0	0	0	0	0	0	0	0	0	Fungi; Ascomycota; Leotiomycetes; Helotiales; Helotiaceae; Tetracladium
0	0	0	0	0	0	0	0	0	0	0	0	Fungi; Ascomycota; Dothideomycetes; Pleosporales; Didymellaceae; Epicoccum
0	0	0	0	0	0	0	0	0	0	0	0	Fungi; Ascomycota; Eurotiomycetes; Chaetothyriales; Trichomeriaceae; Trichomerium
0	0	0	0	0	0	0	0	0	0	0	0	Fungi; Ascomycota; Dothideomycetes; Pleosporales; Phaeosphaeriaceae; Pseudoophiobolus
0	0	0	0	0	0	0	0	0	0	0	0	Fungi; Ascomycota; Leotiomycetes; Erysiphales; Erysiphaceae; Erysiphe
0	0	0	0	0	0	0	0	0	0	0	0	Fungi; Ascomycota; Dothideomycetes; Pleosporales; Pleosporaceae; Pyrenophora
0	0	0	0	0	0	0	0	0	0	0	0	Fungi; Ascomycota; Dothideomycetes; Dothideomycetes; incertae sedis ; Monodictys
0	0	0	0	0	0	0	0	0	0	0	0	Fungi; Ascomycota; Leotiomycetes; Erysiphales; Erysiphaceae; Blumeria
0	0	0	0	0	0	0	0	0	0	0	0	Fungi; Ascomycota; Dothideomycetes; Capnodiales; Neodevriesiaceae; Neodevriesia
0	0	0	0	0	0	0	0	0	0	0	0	Fungi; Ascomycota; Dothideomycetes; Pleosporales; Leptosphaeriaceae; Leptosphaeria
0	0	0	0	0	0	0	0	0	0	0	0	Fungi; Ascomycota; Dothideomycetes; Capnodiales; Mycosphaerellaceae; Septoria

0	0	0	0	0	0	0	0	0	0	0	0	Fungi; Ascomycota; Dothideomycetes; Pleosporales; Phaeosphaeriaceae; Neosetophoma
0	0	0	0	0	0	0	0	0	0	0	0	Fungi; Ascomycota; Dothideomycetes; Capnodiales; Phaeothecaceae; Phaeotheca
0	0	0	0	0	0	0	0	0	0	0	0	Fungi; Ascomycota; Saccharomycetes; Saccharomycetales; Debaryomycetaceae; Debaryomyces
0	0	0	0	0	0	0	0	0	0	0	0	Fungi; Ascomycota; Dothideomycetes; Pleosporales; Melanommataceae; Alpinaria
0	0	0	0	0	0	0	0	0	0	0	0	Fungi; Ascomycota; Dothideomycetes; Pleosporales; incertae sedis; Periconia
0	0	0	0	0	0	0	0	0	0	0	0	Fungi; Ascomycota; Sordariomycetes; Hypocreales; Bionectriaceae; Geosmithia
0	0	0.1814	0	0.5	0	0	0	0.2568	0.0371	0.1809	0.1067	Others

F1F	F2F	F3F	C1F	C2F	C3F	RT1F	RT2F	RT3F	W1F	W2F	W3F	taxonomy
0.5022	0.4722	0.3572	0.4118	0.3460	0.3924	0.4331	0.4817	0.3718	0.4458	0.4250	0.2067	Fungi; Ascomycota; Dothideomycetes; Capnodiales; Cladosporiaceae; Cladosporium
0.0131	0.0139	0.0096	0.0085	0.0162	0.0061	0.0096	0.0142	0.0168	0.0108	0.0022	0.0022	Fungi; Ascomycota; Leotiomycetes; Erysiphales; Erysiphaceae; Podosphaera
0.0630	0.0859	0.0524	0.0693	0.0779	0.0540	0.0641	0.0635	0.0765	0.0520	0.0772	0.0332	Fungi; Ascomycota; Dothideomycetes; Capnodiales; Mycosphaerellaceae; Mycosphaerella
0	0	0	0	0	0	0	0	0	0.0072	0.0019	0.0006	Fungi; Ascomycota; Saccharomycetes; Saccharomycetales; Saccharomycetaceae; Zygosaccharomyces
0.0198	0.0113	0.0111	0.0086	0.0097	0.0067	0.0063	0.0056	0.0092	0.2364	0.2488	0.2407	Fungi; Ascomycota; Eurotiomycetes; Eurotiales; Aspergillaceae; Aspergillus
0.0288	0.0657	0.0347	0.0379	0.0200	0.0407	0.0546	0.0176	0.0607	0.0539	0.0110	0.1054	Fungi; Ascomycota; Eurotiomycetes; Eurotiales; Aspergillaceae; Penicillium
0.0094	0.0237	0.0391	0.0182	0.0167	0.0071	0.0250	0.0077	0.0152	0.0068	0.0124	0.0200	Fungi; Ascomycota; Saccharomycetes; Saccharomycetales; Metschnikowiaceae; Metschnikowia
0.0376	0.0179	0.0511	0.0666	0.0592	0.0641	0.0658	0.1018	0.0863	0.0026	0.0301	0.0255	Fungi; Ascomycota; Sordariomycetes; Hypocreales; Nectriaceae; Fusarium
0.0199	0.0417	0.0349	0.0312	0.0365	0.0413	0.0251	0.0263	0.0360	0.0150	0.0099	0.0080	Fungi; Ascomycota; Dothideomycetes; Capnodiales; Mycosphaerellaceae; Ramularia
0.0466	0.0398	0.0435	0.0518	0.0526	0.0425	0.0379	0.0327	0.0403	0.0168	0.0151	0.0098	Fungi; Ascomycota; Taphrinomycetes; Taphrinales; Taphrinaceae; Taphrina
0.0129	0.0172	0.0658	0.0236	0.0259	0.0279	0.0179	0.0429	0.0155	0.0139	0.0148	0.0054	Fungi; Ascomycota; Dothideomycetes; Pleosporales; Pleosporaceae; Alternaria
0.0249	0.0231	0.0577	0.0264	0.0652	0.0171	0.0514	0.0334	0.0402	0.0293	0.0071	0.0032	Fungi; Ascomycota; Dothideomycetes; Pleosporales; Didymellaceae; Didymella
0.0002	0.0067	0.0001	0.0000	0.0003	0.0080	0.0135	0.0007	0.0052	0.0000	0.0069	0.2684	Fungi; Ascomycota; Leotiomycetes; Thelebolales; Pseudeurotiaceae; Bettsia
0.0144	0.0123	0.0220	0.0627	0.0576	0.0306	0.0261	0.0167	0.0126	0.0170	0.0061	0.0080	Fungi; Ascomycota; Leotiomycetes; Helotiales; Sclerotiniaceae; Monilinia
0.0561	0.0218	0.0159	0.0125	0.0231	0.0144	0.0154	0.0407	0.0142	0.0205	0.0356	0.0117	Fungi; Ascomycota; Dothideomycetes; Dothideales; Aureobasidiaceae; Aureobasidium
0.0255	0.0271	0.0298	0.0301	0.0266	0.0276	0.0344	0.0219	0.0354	0.0165	0.0291	0.0169	Fungi; Ascomycota; Leotiomycetes; Helotiales; Helotiaceae; Tetracladium
0.0154	0.0127	0.0076	0.0117	0.0325	0.0049	0.0272	0.0021	0.0112	0.0013	0.0048	0.0031	Fungi; Ascomycota; Dothideomycetes; Pleosporales; Didymellaceae; Epicoccum
0.0218	0.0039	0.0169	0.0153	0.0142	0.0706	0.0167	0.0113	0.0178	0.0040	0.0037	0.0029	Fungi; Ascomycota; Eurotiomycetes; Chaetothyriales; Trichomeriaceae; Trichomerium
0.0014	0.0004	0.0016	0.0015	0.0017	0.0011	0.0019	0.0010	0.0016	0.0004	0.0024	0.0008	Fungi; Ascomycota; Dothideomycetes; Pleosporales; Phaeosphaeriaceae; Pseudoophiobolus
0.0090	0.0075	0.0067	0.0084	0.0083	0.0128	0.0053	0.0072	0.0135	0.0007	0.0021	0.0007	Fungi; Ascomycota; Leotiomycetes; Erysiphales; Erysiphaceae; Erysiphe
0	0.0001	0	0	0	0	0	0.0022	0	0	0	0	Fungi; Ascomycota; Dothideomycetes; Pleosporales; Pleosporaceae; Pyrenophora
0.0007	0.0010	0.0017	0.0013	0.0008	0.0019	0.0002	0.0013	0.0003	0.0003	0.0026	0.0000	Fungi; Ascomycota; Dothideomycetes; Dothideomycetes; incertae sedis ; Monodictys
0.0005	0.0022	0.0016	0.0043	0.0094	0.0090	0.0065	0.0039	0.0168	0.0017	0.0018	0.0010	Fungi; Ascomycota; Leotiomycetes; Erysiphales; Erysiphaceae; Blumeria
0.0045	0.0090	0.0219	0.0062	0.0091	0.0094	0.0064	0.0024	0.0136	0.0017	0.0117	0.0011	Fungi; Ascomycota; Dothideomycetes; Capnodiales; Neodevriesiaceae; Neodevriesia
0.0055	0.0026	0.0077	0.0019	0.0077	0.0036	0.0046	0.0043	0.0018	0.0067	0.0020	0.0056	Fungi; Ascomycota; Dothideomycetes; Pleosporales; Leptosphaeriaceae; Leptosphaeria
0.0035	0.0019	0.0104	0.0027	0.0012	0.0035	0.0013	0.0012	0.0014	0.0020	0.0011	0.0006	Fungi; Ascomycota; Dothideomycetes; Capnodiales; Mycosphaerellaceae; Septoria
0.0027	0.0023	0.0044	0.0021	0.0055	0.0026	0.0029	0.0026	0.0068	0.0108	0.0084	0.0012	Fungi; Ascomycota; Dothideomycetes; Pleosporales; Phaeosphaeriaceae; Neosetophoma
0.0020	0.0136	0.0042	0.0091	0.0039	0.0061	0.0021	0.0015	0.0032	0.0052	0.0063	0.0024	Fungi; Ascomycota; Dothideomycetes; Capnodiales; Phaeothecaceae; Phaeotheca

0.0010	0.0000	0.0000	0.0001	0.0002	0.0002	0.0001	0.0003	0.0000	0.0000	0.0003	0.0001	Fungi; Ascomycota; Saccharomycetes; Saccharomycetales; Debaryomycetaceae; Debaryomyces
0.0060	0.0026	0.0102	0.0005	0.0031	0.0263	0.0002	0.0034	0.0051	0.0004	0.0004	0.0005	Fungi; Ascomycota; Dothideomycetes; Pleosporales; Melanommataceae; Alpinaria
0.0035	0.0032	0.0010	0.0039	0.0022	0.0107	0.0029	0.0043	0.0052	0.0014	0.0006	0.0006	Fungi; Ascomycota; Dothideomycetes; Pleosporales; incertae sedis; Periconia
0	0	0	0.0001	0	0	0	0.0001	0.0001	0	0	0	Fungi; Ascomycota; Sordariomycetes; Hypocreales; Bionectriaceae; Geosmithia
0.0480	0.0567	0.0791	0.0715	0.0669	0.0571	0.0416	0.0434	0.0657	0.0185	0.0184	0.0137	Others

F1N	F2N	F3N	C1N	C2N	C3N	RT1N	RT2N	RT3N	W1N	W2N	W3N	taxonomy
0.2852	0.3448	0.1793	0.1398	0.2123	0.2065	0.2008	0.1668	0.1768	0.0903	0.1314	0.0748	Fungi; Ascomycota; Dothideomycetes; Capnodiales; Cladosporiaceae; Cladosporium
0.0683	0.1524	0.6562	0.3044	0.2928	0.1976	0.0351	0.1154	0.1335	0.1401	0.1702	0.1557	Fungi; Ascomycota; Leotiomycetes; Erysiphales; Erysiphaceae; Podosphaera
0.1677	0.0924	0.0531	0.1946	0.1863	0.0950	0.2535	0.1904	0.1741	0.0744	0.0930	0.0879	Fungi; Ascomycota; Dothideomycetes; Capnodiales; Mycosphaerellaceae; Mycosphaerella
0.0002	0.0001	0.0001	0.0009	0.0005	0.0007	0.0095	0.0478	0.0193	0.5912	0.4577	0.4776	Fungi; Ascomycota; Saccharomycetes; Saccharomycetales; Saccharomycetaceae; Zygosaccharomyces
0.0021	0.0022	0.0005	0.0018	0.0020	0.0055	0.0007	0.0113	0.0042	0.0161	0.0268	0.0975	Fungi; Ascomycota; Eurotiomycetes; Eurotiales; Aspergillaceae; Aspergillus
0.0614	0.0108	0.0046	0.0118	0.0324	0.0257	0.0105	0.0113	0.0378	0.0010	0.0015	0.0018	Fungi; Ascomycota; Eurotiomycetes; Eurotiales; Aspergillaceae; Penicillium
0.0884	0.0768	0.0070	0.0133	0.0192	0.0407	0.0187	0.2069	0.0178	0.0218	0.0052	0.0104	Fungi; Ascomycota; Saccharomycetes; Saccharomycetales; Metschnikowiaceae; Metschnikowia
0.0011	0.0124	0.0005	0.0024	0.0031	0.0086	0.0061	0.0019	0.0027	0.0041	0.0033	0.0013	Fungi; Ascomycota; Sordariomycetes; Hypocreales; Nectriaceae; Fusarium
0.0455	0.0188	0.0159	0.0208	0.0277	0.0289	0.0404	0.0212	0.0342	0.0067	0.0077	0.0069	Fungi; Ascomycota; Dothideomycetes; Capnodiales; Mycosphaerellaceae; Ramularia
0.0296	0.0158	0.0084	0.0084	0.0077	0.0176	0.0159	0.0075	0.0116	0.0019	0.0213	0.0041	Fungi; Ascomycota; Taphrinomycetes; Taphrinales; Taphrinaceae; Taphrina
0.0565	0.0482	0.0046	0.0291	0.0322	0.0336	0.0290	0.0168	0.0274	0.0041	0.0081	0.0097	Fungi; Ascomycota; Dothideomycetes; Pleosporales; Pleosporaceae; Alternaria
0.0187	0.0197	0.0118	0.0045	0.0089	0.0298	0.0185	0.0056	0.0139	0.0043	0.0183	0.0063	Fungi; Ascomycota; Dothideomycetes; Pleosporales; Didymellaceae; Didymella
0.0004	0.0004	0.0002	0	0	0.0035	0.0050	0	0.1537	0.0001	0.0001	0.0000	Fungi; Ascomycota; Leotiomycetes; Thelebolales; Pseudeurotiaceae; Bettsia
0.0209	0.0279	0.0095	0.0364	0.0320	0.0228	0.0141	0.0176	0.0382	0.0058	0.0047	0.0028	Fungi; Ascomycota; Leotiomycetes; Helotiales; Sclerotiniaceae; Monilinia
0.0126	0.0122	0.0033	0.0054	0.0074	0.0224	0.0104	0.0047	0.0071	0.0021	0.0035	0.0022	Fungi; Ascomycota; Dothideomycetes; Dothideales; Aureobasidiaceae; Aureobasidium
0.0050	0.0019	0.0007	0.0032	0.0013	0.0033	0.0033	0.0025	0.0056	0.0019	0.0020	0.0025	Fungi; Ascomycota; Leotiomycetes; Helotiales; Helotiaceae; Tetracladium
0.0083	0.0039	0.0026	0.0178	0.0050	0.0053	0.0312	0.0040	0.0385	0.0028	0.0044	0.0034	Fungi; Ascomycota; Dothideomycetes; Pleosporales; Didymellaceae; Epicoccum
0.0084	0.0054	0.0011	0.0009	0.0014	0.0145	0.0012	0.0008	0.0026	0.0004	0.0018	0.0007	Fungi; Ascomycota; Eurotiomycetes; Chaetothyriales; Trichomeriaceae; Trichomerium
0.0027	0.0075	0.0006	0.0079	0.0036	0.0050	0.1845	0.0032	0.0061	0.0017	0.0010	0.0016	Fungi; Ascomycota; Dothideomycetes; Pleosporales; Phaeosphaeriaceae; Pseudoophiobolus
0.0221	0.0098	0.0049	0.0080	0.0138	0.0122	0.0187	0.0060	0.0075	0.0011	0.0024	0.0028	Fungi; Ascomycota; Leotiomycetes; Erysiphales; Erysiphaceae; Erysiphe
0	0.0084	0	0.1139	0.0192	0	0.0019	0.0220	0.0006	0	0.0007	0.0011	Fungi; Ascomycota; Dothideomycetes; Pleosporales; Pleosporaceae; Pyrenophora
0.0171	0.0041	0.0013	0.0088	0.0106	0.0830	0.0088	0.0092	0.0060	0.0010	0.0027	0.0046	Fungi; Ascomycota; Dothideomycetes; Dothideomycetes; incertae sedis ; Monodictys
0.0045	0.0282	0.0004	0.0054	0.0062	0.0036	0.0055	0.0061	0.0029	0.0017	0.0025	0.0236	Fungi; Ascomycota; Leotiomycetes; Erysiphales; Erysiphaceae; Blumeria
0.0013	0.0022	0.0006	0.0003	0.0000	0.0021	0.0004	0.0004	0.0005	0.0006	0.0002	0.0002	Fungi; Ascomycota; Dothideomycetes; Capnodiales; Neodevriesiaceae; Neodevriesia
0.0075	0.0039	0.0008	0.0008	0.0017	0.0089	0.0016	0.0012	0.0021	0.0007	0.0007	0.0006	Fungi; Ascomycota; Dothideomycetes; Pleosporales; Leptosphaeriaceae; Leptosphaeria
0.0050	0.0050	0.0017	0.0024	0.0035	0.0093	0.0132	0.0033	0.0030	0.0017	0.0026	0.0009	Fungi; Ascomycota; Dothideomycetes; Capnodiales; Mycosphaerellaceae; Septoria
0.0015	0.0043	0.0008	0.0020	0.0005	0.0050	0.0026	0.0004	0.0061	0.0015	0.0019	0.0001	Fungi; Ascomycota; Dothideomycetes; Pleosporales; Phaeosphaeriaceae; Neosetophoma
0.0027	0.0030	0.0009	0.0026	0.0002	0.0037	0.0011	0.0004	0.0009	0.0005	0.0003	0.0003	Fungi; Ascomycota; Dothideomycetes; Capnodiales; Phaeothecaceae; Phaeotheca

0.0070	0.0082	0.0019	0.0113	0.0113	0.0074	0.0076	0.0097	0.0044	0.0005	0.0005	0.0023	Fungi; Ascomycota; Saccharomycetes; Saccharomycetales; Debaryomycetaceae; Debaryomyces
0	0	0.0000	0.0007	0	0	0	0.0003	0.0000	0.0001	0.0002	0	Fungi; Ascomycota; Dothideomycetes; Pleosporales; Melanommataceae; Alpinaria
0.0008	0.0025	0.0001	0.0004	0.0016	0.0012	0.0006	0.0001	0.0043	0.0037	0.0006	0.0004	Fungi; Ascomycota; Dothideomycetes; Pleosporales; incertae sedis; Periconia
0.0002	0.0015	0.0001	0.0000	0.0011	0.0271	0.0001	0.0362	0.0027	0	0.0001	0	Fungi; Ascomycota; Sordariomycetes; Hypocreales; Bionectriaceae; Geosmithia
0.0475	0.0655	0.0264	0.0400	0.0543	0.0697	0.0496	0.0688	0.0536	0.0162	0.0227	0.0157	Others

Table S4 Calculated relative abundance of bacterial composition (> 10%) between different storage conditions

F1H	F2H	F3H	C1H	C2H	C3H	RT1H	RT2H	W1H	W2H	W3H	taxonomy
0.4335	0.6673	0.8104	0.5924	0.7750	0.6638	0.8669	0.9062	0.52483	0.07480	0.54757	Bacteria;Firmicutes;Bacilli;Lactobacillales;Lactobacillaceae;Lactobacillus;
0.1430	0.0173	0.0255	0.0070	0.0255	0.0864	0.0149	0.0067	0.00106	0	0.01743	Bacteria;Proteobacteria;Gammaproteobacteria;Pseudomonadales;Pseudomonadaceae;Pseudomonas;
0.0256	0.0505	0.0043	0.2206	0.0079	0.0834	0.0022	0.0033	0.02460	0.02002	0.20771	Bacteria;Proteobacteria;Gammaproteobacteria;Pseudomonadales;Moraxellaceae;Acinetobacter;
0.1204	0.0038	0.0084	0.0041	0.0025	0.0064	0.0976	0.0012	0	0	0.03213	Bacteria;Proteobacteria;Gammaproteobacteria;Enterobacteriales;Enterobacteriaceae;Rosenbergiella;
0.0124	0.0117	0.0053	0.0014	0.0032	0.0045	0.0001	0.0006	0.32299	0.75366	0.06831	Bacteria;Actinobacteria;Actinobacteria;Actinomycetales;Micrococcaceae;Arthrobacter;
0.2651	0.2494	0.1462	0.1746	0.1858	0.1554	0.0182	0.0821	0.12653	0.15152	0.12685	Others

F1F	F2F	F3F	C1F	C2F	C3F	RT1F	RT2F	RT3F	W1F	W2F	W3F	taxonomy
0.5824	0.8154	0.7050	0.7270	0.4291	0.6423	0.5668	0.5939	0.6394	0.9061	0.5689	0.8142	Bacteria;Firmicutes;Bacilli;Lactobacillales;Lactobacillaceae;Lactobacillus;
0.1086	0.0126	0.0356	0.0557	0.1557	0.1447	0.1173	0.1578	0.1004	0.0211	0.0641	0.0277	Bacteria;Proteobacteria;Gammaproteobacteria;Pseudomonadales;Pseudomonadaceae;Pseudomonas;
0.0233	0.0041	0.0137	0.0192	0.0062	0.0046	0.0398	0.0150	0.0285	0.0005	0.1711	0.0076	Bacteria;Proteobacteria;Gammaproteobacteria;Pseudomonadales;Moraxellaceae;Acinetobacter;
0.1586	0.0390	0.1071	0.0515	0.0477	0.0603	0.1209	0.0730	0.0275	0.0275	0.0572	0.0423	Bacteria;Proteobacteria;Gammaproteobacteria;Enterobacteriales;Enterobacteriaceae;Rosenbergiella;
0	0	0	0	0	0	0	0	0	0	0	0	Bacteria;Actinobacteria;Actinobacteria;Actinomycetales;Micrococcaceae;Arthrobacter;
0.1271	0.1288	0.1387	0.1466	0.3613	0.1481	0.1551	0.1604	0.2042	0.0448	0.1387	0.1081	Others

F1N	F2N	F3N	C1N	C2N	C3N	RT1N	RT2N	RT3N	W1N	W2N	W3N	taxonomy
0.0479	0.0035	0.0143	0.0459	0.0570	0.0135	0.0015	0.0062	0.0524	0.0348	0.0273	0.0045	Bacteria;Firmicutes;Bacilli;Lactobacillales;Lactobacillaceae;Lactobacillus;
0.3984	0.1424	0.5023	0.3574	0.5189	0.3670	0.2967	0.2924	0.4553	0.1264	0.4771	0.2746	Bacteria;Proteobacteria;Gammaproteobacteria;Pseudomonadales;Pseudomonadaceae;Pseudomonas;
0.2276	0.2436	0.1581	0.1220	0.1436	0.1489	0.2432	0.1671	0.0531	0.3207	0.0647	0.3570	Bacteria;Proteobacteria;Gammaproteobacteria;Pseudomonadales;Moraxellaceae;Acinetobacter;
0.0617	0.3576	0.0128	0.0267	0.0121	0.0183	0.1409	0.0250	0.0128	0.0723	0.0066	0.0719	Bacteria;Proteobacteria;Gammaproteobacteria;Enterobacteriales;Enterobacteriaceae;Rosenbergiella;
0	0	0	0	0	0	0	0	0	0	0	0	Bacteria;Actinobacteria;Actinobacteria;Actinomycetales;Micrococcaceae;Arthrobacter;
0.2645	0.2529	0.3124	0.4480	0.2684	0.4523	0.3177	0.5093	0.4263	0.4458	0.4243	0.2920	Others

Table S5 Calculated relative abundance of fungal composition (> 10%) between different storage conditions

F1H	F2H	F3H	C1H	C2H	C3H	RT1H	RT2H	RT3H	W1H	W2H	W3H	taxonomy
1	1	0.8186	1	0.5	1	1	0.4711	0.7432	0.1104	0.1276	0.5791	Fungi; Ascomycota; Dothideomycetes; Capnodiales; Cladosporiaceae; Cladosporium
0	0	0	0	0	0	0	0.3772	0	0	0	0	Fungi; Ascomycota; Leotiomycetes; Erysiphales; Erysiphaceae; Podosphaera
0	0	0	0	0	0	0	0	0	0	0	0	Fungi; Ascomycota; Dothideomycetes; Capnodiales; Mycosphaerellaceae; Mycosphaerella
0	0	0	0	0	0	0	0.1517	0	0.8525	0.6914	0.3141	Fungi; Ascomycota; Saccharomycetes; Saccharomycetales; Saccharomycetaceae; Zygosaccharomyces
0	0	0	0	0	0	0	0	0	0	0	0	Fungi; Ascomycota; Eurotiomycetes; Eurotiales; Aspergillaceae; Aspergillus
0	0	0.1814	0	0.5	0	0	0	0.2568	0.0371	0.1809	0.1067	Others

F1F	F2F	F3F	C1F	C2F	C3F	RT1F	RT2F	RT3F	W1F	W2F	W3F	taxonomy
0.5022	0.4722	0.3572	0.4118	0.3460	0.3924	0.4331	0.4817	0.3718	0.4458	0.4250	0.2067	Fungi; Ascomycota; Dothideomycetes; Capnodiales; Cladosporiaceae; Cladosporium
0.0131	0.0139	0.0096	0.0085	0.0162	0.0061	0.0096	0.0142	0.0168	0.0108	0.0022	0.0022	Fungi; Ascomycota; Leotiomycetes; Erysiphales; Erysiphaceae; Podosphaera
0.0630	0.0859	0.0524	0.0693	0.0779	0.0540	0.0641	0.0635	0.0765	0.0520	0.0772	0.0332	Fungi; Ascomycota; Dothideomycetes; Capnodiales; Mycosphaerellaceae; Mycosphaerella
0	0	0	0	0	0	0	0	0	0.0072	0.0019	0.0006	Fungi; Ascomycota; Saccharomycetes; Saccharomycetales; Saccharomycetaceae; Zygosaccharomyces
0.0198	0.0113	0.0111	0.0086	0.0097	0.0067	0.0063	0.0056	0.0092	0.2364	0.2488	0.2407	Fungi; Ascomycota; Eurotiomycetes; Eurotiales; Aspergillaceae; Aspergillus
0.4019	0.4166	0.5697	0.5018	0.5502	0.5409	0.4869	0.4351	0.5256	0.2477	0.2449	0.5166	Others

F1N	F2N	F3N	C1N	C2N	C3N	RT1N	RT2N	RT3N	W1N	W2N	W3N	taxonomy
0.2852	0.3448	0.1793	0.1398	0.2123	0.2065	0.2008	0.1668	0.1768	0.0903	0.1314	0.0748	Fungi; Ascomycota; Dothideomycetes; Capnodiales; Cladosporiaceae; Cladosporium
0.0683	0.1524	0.6562	0.3044	0.2928	0.1976	0.0351	0.1154	0.1335	0.1401	0.1702	0.1557	Fungi; Ascomycota; Leotiomycetes; Erysiphales; Erysiphaceae; Podosphaera
0.1677	0.0924	0.0531	0.1946	0.1863	0.0950	0.2535	0.1904	0.1741	0.0744	0.0930	0.0879	Fungi; Ascomycota; Dothideomycetes; Capnodiales; Mycosphaerellaceae; Mycosphaerella
0.0002	0.0001	0.0001	0.0009	0.0005	0.0007	0.0095	0.0478	0.0193	0.5912	0.4577	0.4776	Fungi; Ascomycota; Saccharomycetes; Saccharomycetales; Saccharomycetaceae; Zygosaccharomyces
0.0021	0.0022	0.0005	0.0018	0.0020	0.0055	0.0007	0.0113	0.0042	0.0161	0.0268	0.0975	Fungi; Ascomycota; Eurotiomycetes; Eurotiales; Aspergillaceae; Aspergillus
0.4766	0.4080	0.1109	0.3584	0.3061	0.4949	0.5004	0.4683	0.4921	0.0878	0.1209	0.1064	Others

Table S6 Results of statistical analysis 1 (GLM) for bacterial genera in Dataset 2 (additionally Dataset 1) (> 10%)

**GLM (Dataset 2-2019 (F and N))**	**df1**	**df2**	**F.ratio**	**p.value**
**Lactobacillus**				
Location	1	lnf	524.107	<0.0001
Storage condition	3	lnf	2.792	0.0388
Location:Storage condition	3	lnf	3.014	0.0187
**Pseudomonas**				
Location	1	lnf	61.793	<0.0001
Storage condition	3	lnf	2.303	0.0748
Location:Storage condition	3	lnf	0.338	0.798
**Acinetobacter**				
Location	1	lnf	43.311	<0.0001
Storage condition	3	lnf	0.602	0.6138
Location:Storage condition	3	lnf	0.916	0.4319
**Rosenbergiella**				
Location	1	lnf	1.249	0.2637
Storage condition	3	lnf	0.954	0.4132
Location:Storage condition	3	lnf	0.132	0.941

**GLM (Dataset 1&2- 2018&2019 (H, F and N))**	**df1**	**df2**	**F.ratio**	**p.value**
**Lactobacillus**				
Location	2	lnf	260.289	<0.0001
Storage condition	3	lnf	2.378	0.0677
Location:Storage condition	6	lnf	7.032	<0.0001
**Pseudomonas**				
Location	2	lnf	62.422	<0.0001
Storage condition	3	lnf	2.85	0.0359
Location:Storage condition	6	lnf	1.296	0.2551
**Acinetobacter**				
Location	2	lnf	18.475	<0.0001
Storage condition	3	lnf	0.667	0.5724
Location:Storage condition	6	lnf	1.096	0.3616
**Rosenbergiella**				
Location	2	lnf	7.616	0.0005
Storage condition	3	lnf	1.266	0.2842
Location:Storage condition	6	lnf	0.214	0.9724
**Arthrobacter**				
Location	2	lnf	9.421	0.0001
Storage condition	3	lnf	5.425	0.001
Location:Storage condition	6	lnf	2.821	0.0096

Table S7 Results of statistical analysis 2 (Tukey Test) for bacterial genera in Dataset 2 (additionally Dataset 1) (> 10%)

**Tukey Test (Dataset 2-2019 (F and N))**	estimate	SE	df	z.ratio	p.value
**Lactobacillus**					
cold-fresh	-0.0404	0.0396	lnf	-1.02	0.7378
cold-room temp	0.0157	0.0407	lnf	0.386	0.9805
cold-warm	-0.0815	0.038	lnf	-0.2145	0.1391
fresh-room temp	0.0561	0.0387	lnf	1.45	0.4683
fresh-warm	-0.0412	0.0358	lnf	-1.15	0.6587
room temp-warm	-0.0972	0.0371	lnf	-2.623	0.0432

**Tukey Test (Dataset 1&2-2018&2019 (H, F and N))**	estimate	SE	df	z.ratio	p.value
**Lactobacillus**					
F-H	0.0381	0.0463	lnf	0.822	0.6892
F-N	0.6235	0.0356	lnf	17.516	<0.0001
H-N	0.5854	0.0348	lnf	16.828	<0.0001
**Pseudomonas**					
cold-fresh	0.0464	0.0309	lnf	1.505	0.4347
cold-room temp	0.0338	0.0321	lnf	0.71	0.893
cold-warm	0.0806	0.0294	lnf	2.745	0.0308
fresh-room temp	-0.0236	0.0305	lnf	-0.773	0.8667
fresh-warm	0.0342	0.0276	lnf	1.237	0.603
room temp-warm	0.0578	0.029	lnf	1.99	0.1917
F-H	0.0575	0.0172	lnf	3.339	0.0024
F-N	-0.2525	0.0307	lnf	-8.223	<0.0001
H-N	-0.3099	0.0281	lnf	-11.047	<0.0001
**Acinetobacter**					
F-H	-0.0178	0.0143	lnf	-1.246	0.436
F-N	-0.01549	0.0255	lnf	-6.078	<0.0001
H-N	-0.01371	0.0268	lnf	-5.12	<0.0001
**Rosenbergiella**					
F-H	0.0568	0.0165	lnf	3.445	0.0017
F-N	0.0212	0.0194	lnf	1.094	0.5175
H-N	-0.0356	0.0142	lnf	-2.513	0.0321
**Arthrobacter**					
cold-fresh	-0.00407	0.00993	lnf	-0.41	0.9767
cold-room temp	0.00351	0.00812	lnf	0.432	0.9729
cold-warm	-0.10362	0.0269	lnf	-3.853	0.0007
fresh-room temp	0.00758	0.00949	lnf	0.799	0.8548
fresh-warm	-0.09955	0.02733	lnf	-3.642	0.0015
room temp-warm	-0.10713	0.02671	lnf	-4.012	0.0004
F-H	-0.0908	0.0211	lnf	-4.302	0.0001
F-N	0	0.0056	lnf	0	1
H-N	0.0908	0.0211	lnf	4.302	0.0001

Table S8 Results of statistical analysis 1 (GLM) for fungal genera in Dataset 2 (> 10%)

**GLM (Dataset 2-2019 (F and N))**	df1	df2	F.ratio	p.value
**Cladosporium**				
Location	1	Inf	95.146	<0.0001
Storage condition	3	Inf	6.009	0.0004
Location:Storage condition	3	Inf	0.642	0.5879
**Podoshaera**				
Location	1	Inf	40.318	<0.0001
Storage condition	3	Inf	2.218	0.0837
Location:Storage condition	3	Inf	2.389	0.0668
**Mycosphaerella**				
Location	1	Inf	45.613	<0.0001
Storage condition	3	Inf	8.078	<0.0001
Location:Storage condition	3	Inf	5.337	0.0011
**Zygosaccharomyces**				
Location	1	Inf	643.042	<0.0001
Storage condition	3	Inf	221.964	<0.0001
Location:Storage condition	3	Inf	217.176	<0.0001
**Aspergillus**				
Location	1	Inf	139.089	<0.0001
Storage condition	3	Inf	72.087	<0.0001
Location:Storage condition	3	Inf	41.366	<0.0001

Table S9 Results of statistical analysis 2 (Tukey Test) for fungal genera in Dataset 2 (> 10%)

**Tukey Test (Dataset 2-2019 (F and N))**	estimate	SE	df	z.ratio	p.value
**Cladosporium**					
cold-fresh	-0.0688	0.0325	Inf	-2.114	0.1486
cold-room temp	-0.021	0.0316	Inf	-0.664	0.9106
cold-warm	0.0595	0.0299	Inf	1.988	0.1926
fresh-room temp	0.0478	0.0327	Inf	1.463	0.46
fresh-warm	0.1283	0.0311	Inf	4.126	0.0002
room temp-warm	0.0808	0.0301	Inf	2.675	0.0375
**Mycosphaerella**					
cold-fresh	0.0288	0.0146	Inf	1.968	0.2
cold-room temp	-0.0263	0.0163	Inf	-1.621	0.3667
cold-warm	0.041	0.0142	Inf	2.893	0.0199
fresh-room temp	-0.0552	0.0154	Inf	-3.589	0.0019
fresh-warm	0.0122	0.0132	Inf	0.927	0.7903
room temp-warm	0.0674	0.0149	Inf	4.512	<0.0001
**Zygosaccharomyces**					
cold-fresh	0.000471	0.000884	Inf	0.533	0.9511
cold-room temp	-0.010296	0.002941	Inf	-3.499	0.0026
cold-warm	-0.254761	0.009996	Inf	-25.485	<0.0001
fresh-room temp	-0.010767	0.002908	Inf	-3.702	0.0012
fresh-warm	-0.255232	0.009985	Inf	-25.562	<0.0001
room temp-warm	-0.244466	0.010374	Inf	-23.565	<0.0001
**Aspergillus**					
cold-fresh	-0.001901	0.0031	Inf	-0.613	0.9279
cold-room temp	0.000427	0.00281	Inf	0.152	0.9987
cold-warm	-0.132687	0.00916	Inf	-14.488	<0.0001
fresh-room temp	0.002328	0.00305	Inf	0.764	0.8708
fresh-warm	-0.0130785	0.00925	Inf	-14.161	<0.0001
room temp-warm	-0.0133114	0.00914	Inf	-14.562	<0.0001

Figure S1 Location area of sampling in Southern Germany, Baden-Wuerttemberg with different colors showing the different altitude regions. (Mapping with JMP® pro 15.0; basic data from the Environmental Information System (UIS) of the LUBW State Institute for the Environment Baden-Wuerttemberg and basic data © State Office for Geoinformation and Rural Development Baden-Wuerttemberg (www.lgl-bw.de))

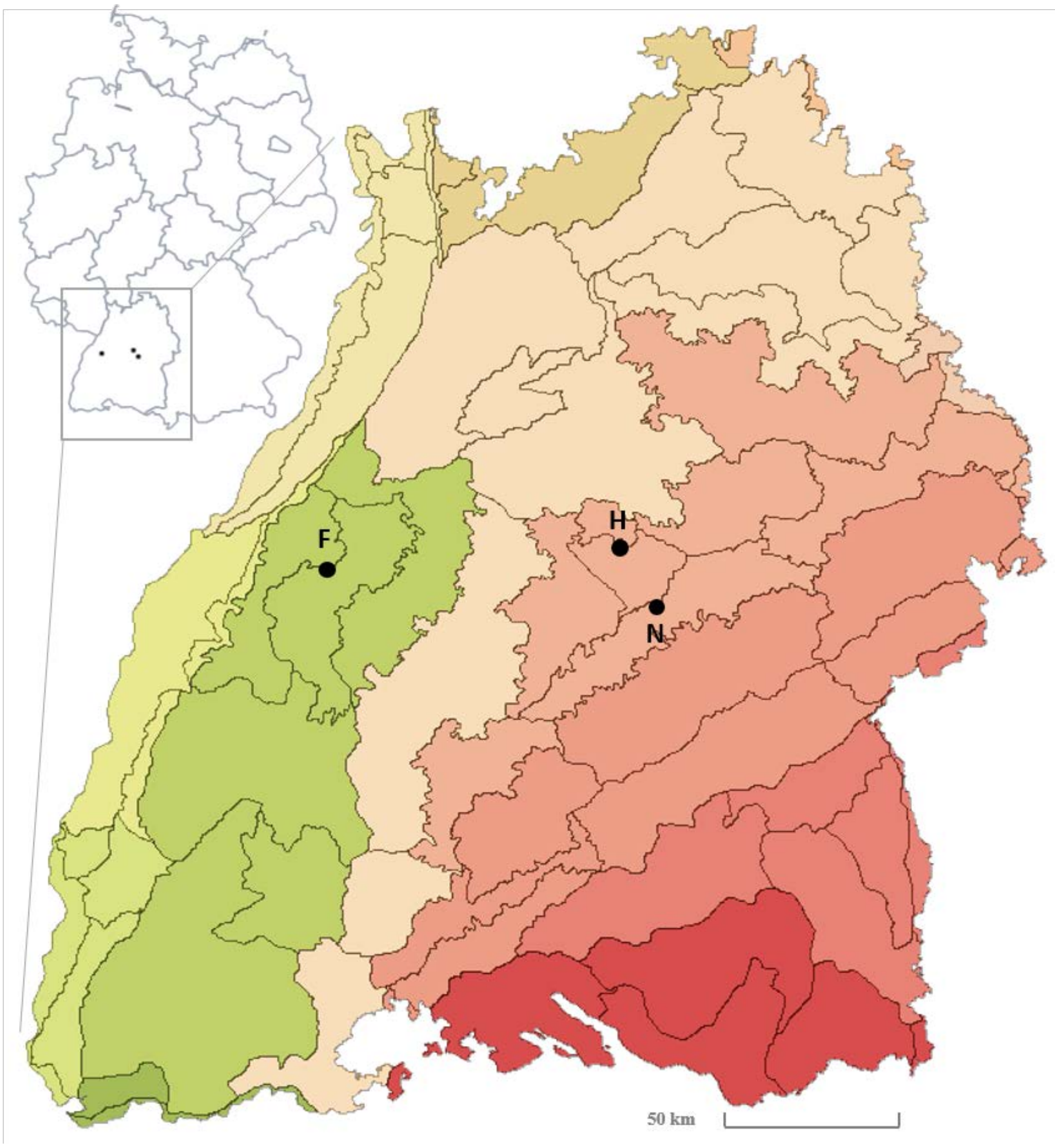

Figure S2 Storage cabinet with moisture meter and saturated sodiumchloride solution (“warm” storage condition)

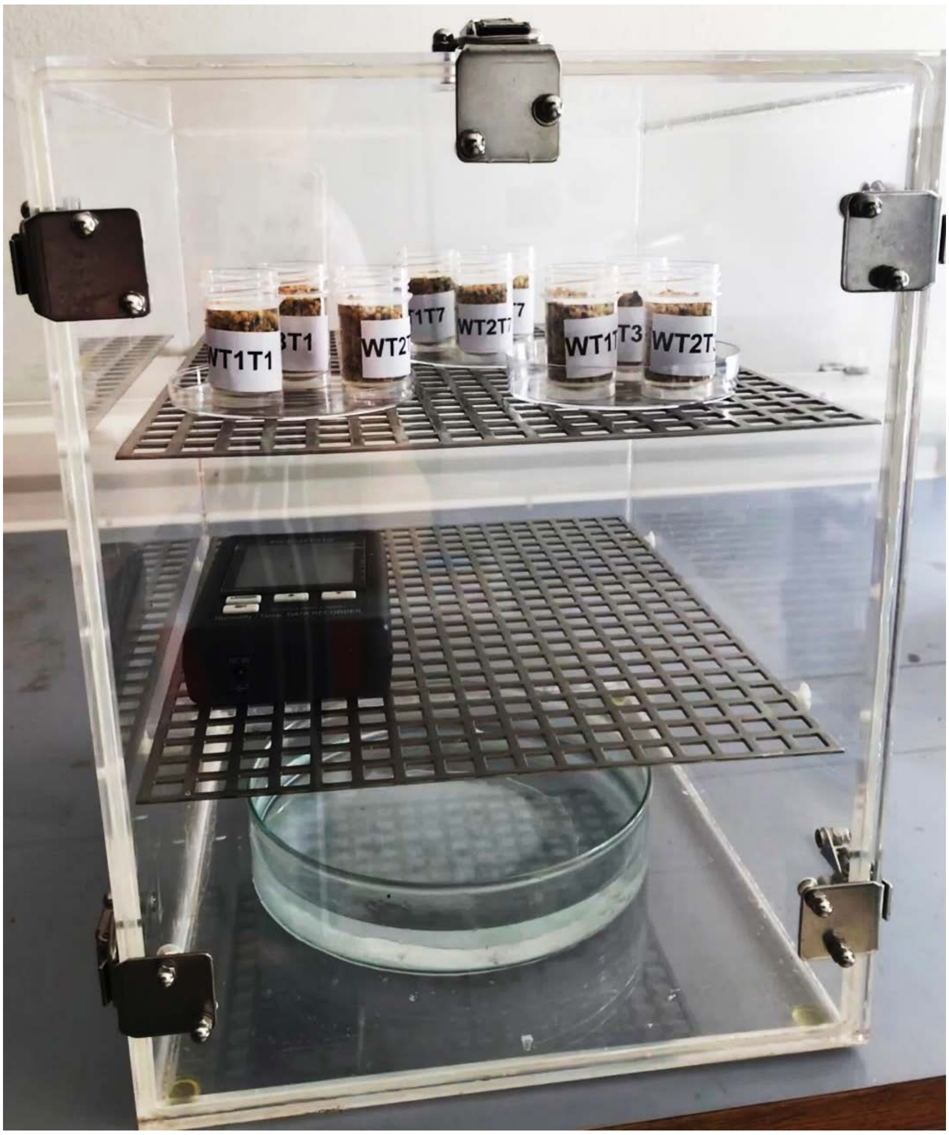

Figure S3 Stack bar chart, showing the composition of bacterial (a) and fungal (b) communities of Dataset 1 (H) and Dataset 2 (F and N) (filtered on minimum of 10% average) in fresh and stored bee pollen

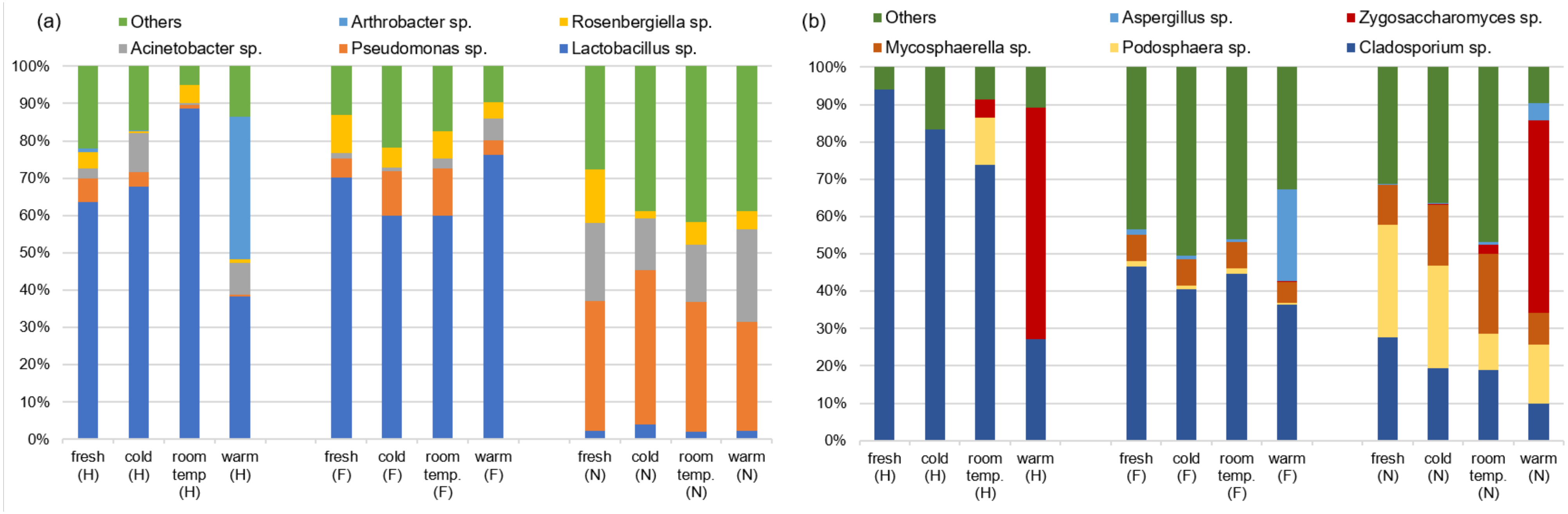

Supplement: Supplementary file 1 — (PDF 598 kb). [file 11356_2021_13932_MOESM1_ESM.pdf]
